# Time-resolved monitoring of yeast responses to lipopolysaccharide exposure by cell-released volatile organic compounds

**DOI:** 10.1128/aem.00785-25

**Published:** 2025-08-06

**Authors:** Huaying Liu, Maosheng Yao

**Affiliations:** 1 State Key Joint Laboratory of Environmental Simulation and Pollution Control, College of Environmental Sciences and Engineering, Peking University 364600, Beijing, China; Chalmers tekniska hogskola AB, Gothenburg, Sweden

**Keywords:** *Saccharomyces cerevisiae*, lipopolysaccharide, volatile organic compounds, proteomics, transcriptomics, metabolism

## Abstract

Volatile organic compound (VOC) profiles function as dynamic fingerprints of physiological states and disease progression. Here, the eukaryotic organism *Saccharomyces cerevisiae* was used to investigate the emission characteristics of VOCs induced by lipopolysaccharide (LPS). Using multi-omics techniques and physiology methods, yeast cells were observed to undergo both stress and adaptation phases upon exposure, as characterized by changes in acetic acid-D and higher alcohols/aldehydes. The oxidative phosphorylation process in yeast was inhibited during the stress response, leading to an oxidative stress accompanied by growth inhibition and cell wall remodeling. The adaptive stage of cells reprogrammed metabolism to consume excess metabolic substrates generated during the stress stage, thus resulting in the production of secondary metabolites such as higher alcohols/aldehydes as biomarkers. Acetic acid detected could, in contrast, serve as an early biomarker for the oxidative stress of yeast. Using flow cytometry together with FITC labeling, LPS was further shown to bind to cells, leading to internalization and membrane damage compared to controls. This study provides time-resolved mechanistic insights into VOCs as non-invasive biomarkers. These findings suggest that dynamic VOC profiles from cells hold promise as a “surveillance camera” for monitoring cellular health.

**IMPORTANCE:**

The analysis of metabolically derived volatile organic compounds (VOCs) provides an approach for tracking cellular stress dynamics. We demonstrate that the VOC profile released by *S. cerevisiae* cells dynamically evolved with time during lipopolysaccharide (LPS)-induced stress, coordinated with transcriptomic and proteomic reprogramming. Through multiple omics techniques and physiology, yeast cells were observed to undergo both stress and adaptation phases, as characterized by changes in acetic acid-D and higher alcohols/aldehydes. These findings establish VOCs as real-time, non-invasive indicators of time-resolved stress responses, highlighting their potential as surveillance tools to detect early cellular perturbations caused by external threats.

## INTRODUCTION

The cellular stress response is a vital component of organisms’ defense mechanisms against toxic insults (1). Generally, it is understood as the adaptive defense mechanisms by which cells respond to environmental pollutants in various ways, including oxidative stress, DNA damage response, autophagy, and cell death (2–5). Prolonged pollutant exposure induces functional and structural disorders at the molecular level, which correspondingly leads to cell aging and programmed death in human diseases such as degenerative diseases and tumors (6). Distinct pollutants activate specialized stress pathways. For example, heavy metal ions represented by iron (Fe) and copper (Cu) are transported into cells by membrane proteins, which form reactive oxygen species (ROS) through Fenton and Haber-Weiss reactions, thereby inducing cellular oxidative stress responses (7). Thus, a detailed understanding (e.g., time-resolved) of the cellular response to exposure could facilitate early detection of subclinical physiological perturbations or incipient disease states.

Lipopolysaccharide (LPS), a common airborne pollutant, is recognized by toll-like receptors on mammalian cell surfaces or intercellular receptors. This interaction activates an immune response that releases inflammatory cytokines, ultimately inducing cellular pyroptosis and apoptosis (8, 9). Inhalation of LPS, which is often bound to dust and atmospheric particulate matter (10), can cause acute airway inflammation and elevate the risk of diseases like asthma (11). Previously, *Saccharomyces cerevisiae*, a well-established disease model, has been used for toxicological research on environmental pollutants, including heavy metals, rare earth elements, nanoparticles, and pesticides, with insights into their signaling and regulatory mechanisms (12–15). Several studies demonstrate that LPS exposure activates autophagy in *S. cerevisiae* via phosphorylation of the mitogen-activated protein (MAP) kinase HOG1 (16, 17). Despite the progress in understanding cell-LPS interactions, time-resolved cell responses to the pollutant exposure and early biological events before a visible biomarker increase are poorly understood at the molecular level. The genetic simplicity and single-cell nature of *S. cerevisiae* make it a powerful model for identifying LPS-induced biomarkers and elucidating stress response mechanisms relevant to mammalian cells.

Identifying early signs of cellular stress response is critical for preventing adverse health effects caused by exposure to foreign substances. Biomarkers serve as valuable early warning indicators to assess an organism’s sensitivity to pollutants. At molecular and biochemical levels, biomarkers reflect rapid organismal responses triggered by pollutants (18). Recently, volatile organic compounds (VOCs) released by cells have gained attention as non-invasive gaseous biomarkers (19, 20). VOCs are cellular metabolites regulated by environmental conditions and biological state, detectable in cultures of multicellular organisms, algae, and yeast (21–23). These compounds are proposed to act as signaling molecules mediating inter-organism communication, conveying stress and defense signals (24, 25). Previously, cumulative VOCs released from cells after exposure to stress components were collected by headspace sampling techniques. For example, *S. cerevisiae* showed oxidative stress after exposure to the cellular H_2_O_2_, O_3_, and CO_2_ (23). The metabolism of *S. cerevisiae* was reprogrammed to release VOCs represented by ethyl acetate for cellular detoxification processes (23). Overall, the VOCs released by cells can reflect the health status of the cells. Furthermore, the evolution of the VOC profile may serve as an important indicator of time-resolved cellular responses to external stimuli, thereby providing insights into early signs of environmental exposure to prevent harm.

This study aimed to determine whether time-resolved VOC profiles can be employed to characterize the diverse processes involved in the biological response to LPS stress. Here, we used the eukaryotic model organism *S. cerevisiae* and LPS to investigate time-resolved cellular events when exposed to pollutants. Using the developed protocol, the time-resolved release of VOCs from living cells during cellular stress by LPS was monitored. Multi-omics integration (transcriptomics, proteomics, and metabolomics) revealed molecular-level regulatory networks activated by LPS exposure. The results of this study provide mechanistic insights into time-resolved cellular responses to stress and hold values for detecting early signs of foreign insults, supporting strategies to mitigate disease progression.

## RESULTS AND DISCUSSION

### *S. cerevisiae* membrane interaction, growth, and budding after LPS exposure

**Fig 1:**
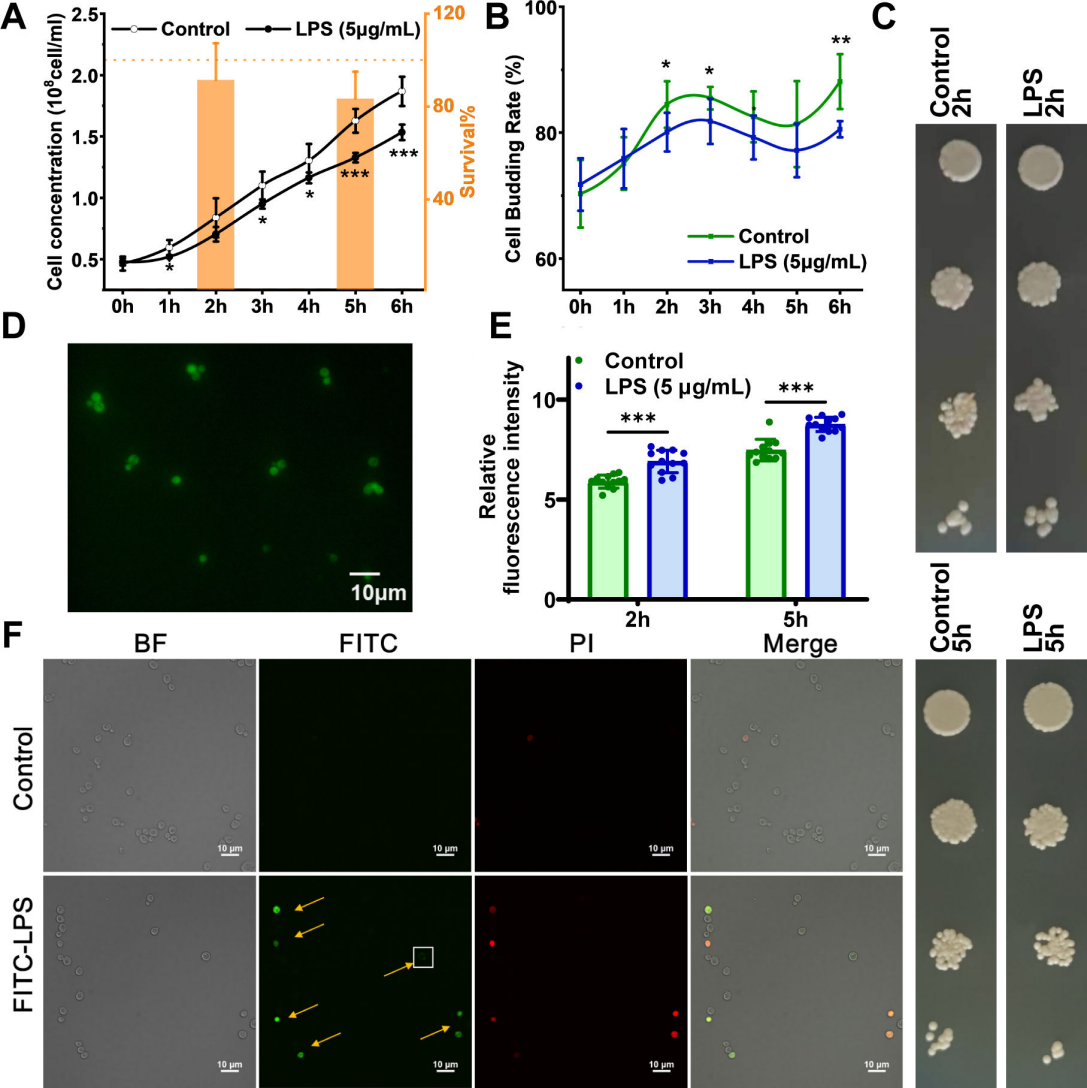
Stress responses and interactions of *S. cerevisiae* in response to LPS. (**A**) Cell viability assay including growth curves and colony-forming units (CFU). The black hollow and solid lines are the growth curves of the control and LPS groups, respectively. Orange represents the survival rate (%) = CFU from LPS group/ CFU from the control group ×100%. (**B**) Cell budding rate curve. Cell budding rate = budding cells / all cells × 100%. (**C**) Spot assays were performed after 2 hours and 5 hours of exposure to LPS. (**D**) Visualization of cellular ROS induced by LPS exposure. (**E**) Quantification of cellular ROS induced by LPS exposure. (**F**) Visualization of cells after incubation with 10 µg/mL FITC-LPS for 1 hour. The control group was treated with an equal volume of PBS. Orange arrows and white boxes indicate the aggregation of FITC-LPS. Data represent mean ± SEM (independent experiment *n* = 6). *P < 0.05, **P < 0.01, and ***P < 0.001 (*t*-test).

Alterations in multiple physiological phenotypes were observed in *S. cerevisiae* after exposure to LPS. The hourly cellular concentration of *S. cerevisiae* revealed that the growth and emergence of *S. cerevisiae* were significantly and consistently inhibited over time after exposure to LPS (Fig. 1A and B). The results of the colony-forming unit and spot assays after 5 hours of exposure to LPS also showed that LPS reduced the ability of the cells to grow and proliferate (Fig. 1A and C). The capacity of LPS to induce oxidative stress in yeast remains debated (16, 26), which may be related to different experimental conditions for different studies. We measured cellular endogenous ROS levels with DCFH-DA ROS probes. Fluorescence quantitative results showed that LPS-induced intracellular ROS levels were increased (Fig. 1D and E). To track the interaction between LPS and yeast cells and determine their adsorption or internalization characteristics, we used fluorescein isothiocyanate-labeled LPS (FITC-LPS) in conjunction with the membrane integrity probe propidium iodide (PI) for tracking analysis. Flow cytometry data showed that FITC-LPS could bind to yeast cells (Fig. S1). Furthermore, LPS exposure caused damage to the cell membrane, which was consistent with findings of previous research (26). The control group exposed solely to FITC indicated that FITC could bind to yeast cells, as detected by flow cytometry. In previous studies, they have shown the binding occurs with the cell wall and vacuoles of healthy cells (27, 28). However, compared to FITC-LPS, FITC itself does not alter the physiological state of yeast cells. Using laser scanning confocal microscopy (Fig. 1F), we have shown that LPS indeed bound to the yeast cells. The Z-stack images (Fig. S2) further substantiated the binding of FITC-LPS to cells, demonstrating either localized fluorescent patches on the cell surface or whole-cell fluorescence distribution. This suggests that the transmembrane transport in yeast cells may have changed, similar to that of mammalian cells. LPS has previously been shown to alter mammalian vesicular transport by internalizing LPS through endosomal vesicles and releasing extracellular vesicles (29–31). In yeast, vesicles contain chitin synthase and cell wall remodeling enzymes and can participate in the transport of large molecules on the cell wall and cell wall remodeling (32–34).

### Time-resolved analysis of VOC release, differentially expressed metabolites (DEMs), and enrichment metabolic pathway

**Fig 2:**
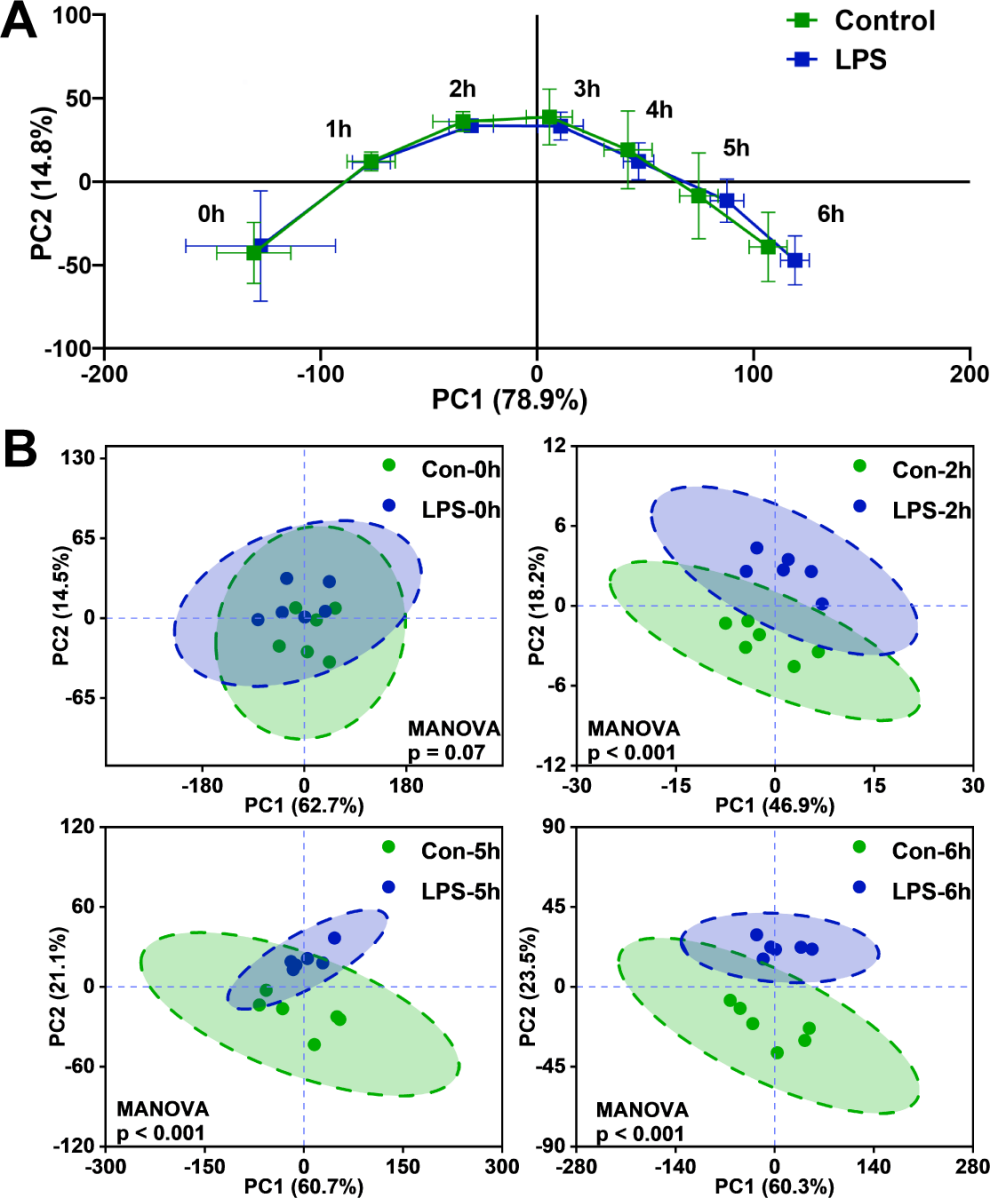
Profiles of extracellular VOC release in *S. cerevisiae* after LPS exposure. (**A**) Time-dependent trajectory of the extracellular VOC metabolic profiles. (**B**) Principal component analysis of VOCs in samples at 0 hours, 2 hours, 5 hours, and 6 hours. Scatter points represent biological replicate samples, and ellipses represent 95% confidence intervals. *P* values are derived from multivariate analysis of variance (MANOVA).

**Fig 3:**
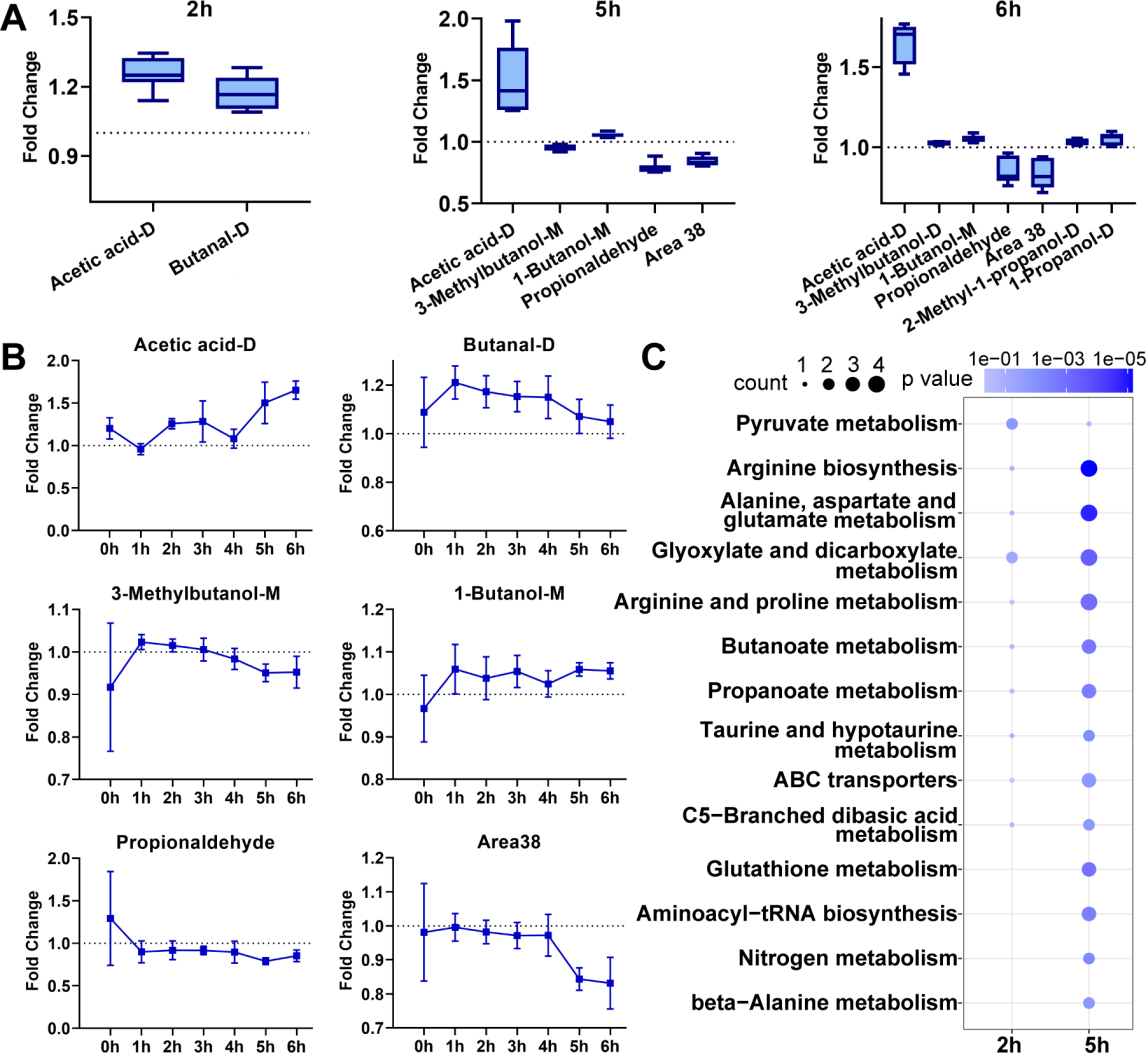
Time-resolved analysis of the release of extracellular VOCs and metabolic reprogramming in *S. cerevisiae* after exposure to LPS. (**A**) Differentially expressed VOCs in 2 hour, 5 hour, and 6 hour samples. The vertical axis denotes the ratio of signal values in samples from treatment and control groups. (**B**) The trend in the release of VOCs over time. (**C**) The KEGG enrichment metabolic pathways were affected by yeast cells exposed to LPS for different periods of time.

Although traditional cell growth assessments and oxidative stress analyses indicate physiological changes in yeast cells, they cannot address the dynamic processes of the cell response to LPS stimulation. To address this limitation, we characterized the metabolic changes in yeast stress response through time-resolved VOC characteristic analysis. A total of 51 peaks of VOC were detected, including VOC monomers (VOC-M), dimers (VOC-D), and peaks of undetermined but highly expressed compounds (Table S1). We measured VOCs for 0–6 hours and analyzed them hourly using principal component analysis (PCA). PCA is an unsupervised analysis method, which helps detect the intrinsic clustering of samples and identify the biological characteristics of grouped samples. To differentiate VOC release characteristics between normal and stressed cells, temporal trajectories of mean principal component scores demonstrated that LPS-exposed and control groups showed indistinguishable VOC patterns initially (Fig. 2A), but exhibited significant separation after 2, 5, and 6 hours of exposure (*P* < 0.001, MANOVA) (Fig. 2B; Fig. S3). Analyses such as *t*-test and partial least squares discriminant analysis (PLS-DA) model on the VOCs of different experimental groups were employed to identify the primary differentially expressed VOC species. Following a 2-hour exposure to LPS in *S. cerevisiae*, the upregulated VOCs were acetic acid-D and butanal-D (Fig. 3A). After being exposed to LPS for 5 hours, *S. cerevisiae* was found to upregulate VOCs such as acetic acid-D, 1-butanol-M, and 3-methylbutanol-D, while downregulating area38 and propionaldehyde (Fig. 3A). The significantly regulated VOCs in the sample after 6 hours of exposure were very similar to those in the 5-hour sample (Fig. 3A), and there was an increase in the release of two additional higher alcohols. Based on these findings, it was determined that the metabolic state of the yeast remained consistent after 5 hours and after 6 hours of exposure. Consequently, our results tentatively suggested that VOC composition changed after 2 and 5 hours of exposure to LPS. Samples from these two time points were selected for further analysis. Compared with the control group, the release of various VOCs exhibited significant changes at different time points, which could be due to the metabolic status of the cells. While the absolute up or downregulation of differentially expressed VOCs in the exposure group was not substantial, it did yield statistically significant results, as analyzed using the *t*-test and partial least squares-discriminant analysis. The trend of upregulation of acetic acid-D, 3-methylbutanol-D, and 1-butanol-M released by *S. cerevisiae* became increasingly evident over time after exposure to LPS (Fig. 3B). For propionaldehyde and area38, a downward trend was observed over time, but the phenomenon of significant weakening was not captured until 5 hours of LPS exposure (Fig. 3B). These VOCs are oxidative or reducing products of different metabolic patterns, implying time-resolved changes in the metabolic state of yeast cells.

To investigate VOC metabolic dynamics under cellular stress, we analyzed the cellular metabolic profile. We identified 36 and 18 intracellular DEMs that can be annotated by the Kyoto Encyclopedia of Genes and Genomes (KEGG) in the samples treated for 2 and 5 hours, respectively (Table S2). We found that these intracellular and extracellular DEMs are associated with multiple metabolic pathways, according to our metabolic pathway enrichment analysis (Fig. 3C). After 2 hours of LPS treatment, yeast cells modified the pyruvate metabolic pathway within yeast cells, which plays a vital role in carbohydrate metabolism. This alteration is mainly due to the amplified expression of acetic acid-M and D-lactate, which are metabolites in the pyruvate metabolic pathway (35). Acetic acid is a well-known volatile organic compound produced by carbohydrate metabolism in yeast cells, which can reflect changes in cellular redox levels and energy metabolism (36). It was shown that NADPH and NADH produced during the synthesis of acetic acid could balance the excess NAD produced during glycerol formation under hyperosmotic stress (37, 38). Carbohydrates provide a crucial cellular energy source. The 2-hour sample reveals that several DEMs are linked to carbohydrate metabolism, including 1-butanal, citric acid, D-galactose, gluconic acid, phthalic acid, and 3-phosphoglycolic acid (Table S2). While no alterations in intracellular pyruvate concentration were detected, it is worth exploring whether LPS exposure can affect energy metabolism. The DEMs of the 5-hour sample involve not only carbohydrate metabolism but also various nitrogen metabolism pathways such as several amino acid synthesis pathways, amino acid metabolism pathways, and glutathione metabolism. Notably, three Ehrlich pathway-derived VOCs (3-methylbutanol-D, 1-butanol-M, and propionaldehyde)—classified as higher alcohols/aldehydes—emerged from α-keto acid decarboxylation (39). Twenty amino acid metabolites showed significant changes (Table S3), with VOC-amino acid correlations (Fig. S4) revealing alcohol-positive and aldehyde-negative associations, consistent with Ehrlich pathway dynamics (40). Amino acid-driven metabolic rewiring suggests substrate prioritization for energy replenishment. Concurrently, glutathione pathway DEMs (L-glutamic acid, ornithine, and pyroglutamic acid) were upregulated (Table S3), implying enhanced ROS defense via glutathione synthesis (41, 42). Yeast cells exhibited distinct metabolic patterns following 2 and 5 hours of LPS exposure, signifying yeast’s metabolic reprogramming in response to LPS stimulation.

### Cellular defense processes, including stress and adaptive responses

Multi-omics analysis, which includes proteomic, transcriptomic, and metabolomic analyses, can provide comprehensive information on the cellular response (43, 44). To investigate the biological implications of time-resolved differences in VOC release, we used a multi-omics approach to probe the defense processes of yeast cells in response to LPS exposure. The results illustrate that yeast cells are capable of establishing robust and rapid responses at a molecular level with limited exposure time (Fig. S5). In the gene or protein expression profile, several time-dependent clusters were observed using the Short Time-series Expression Miner (STEM) program, which showed significant enrichment after false discovery rate correction (*P* value < 0.05) (Fig. S6). The gene (protein) expression in gene cluster 10 and protein cluster 5 returned to the same level as the control group after rapid response to LPS stimulation, suggesting that the yeast cells have an adaptive response to LPS. The Gene Ontology (GO) enrichment results of these gene and protein clusters demonstrated the activation of related regulatory networks within yeast cells. Combined with cell physiological analysis (viability, ROS, and LPS interaction), the stress responses in *S. cerevisiae* toward LPS could entail cell wall-related processes, oxidation-reduction process, and growth regulation. Similar results were obtained for subcellular localization analysis of differentially expressed proteins (DEPs) (Fig. S7) and GO enrichment analysis of DEPs and differentially expressed genes (DEGs) (Fig. S8). For the possible regulatory pathways involved, gene expression and protein levels were further investigated using gene enrichment analysis (GSEA) and heat mapping, respectively. Protein synthesis and degradation are regulated by factors such as exposure time and stress response processes, resulting in different DEPs under different exposure times. In general, yeast cells were under stress after 2 hours of exposure, showing upregulation and downregulation of specific protein levels, while stress was relieved after 5 hours of exposure. Accordingly, in this work, we thoroughly studied the cellular responses after 2-hour and 5-hour exposures.

**Fig 4:**
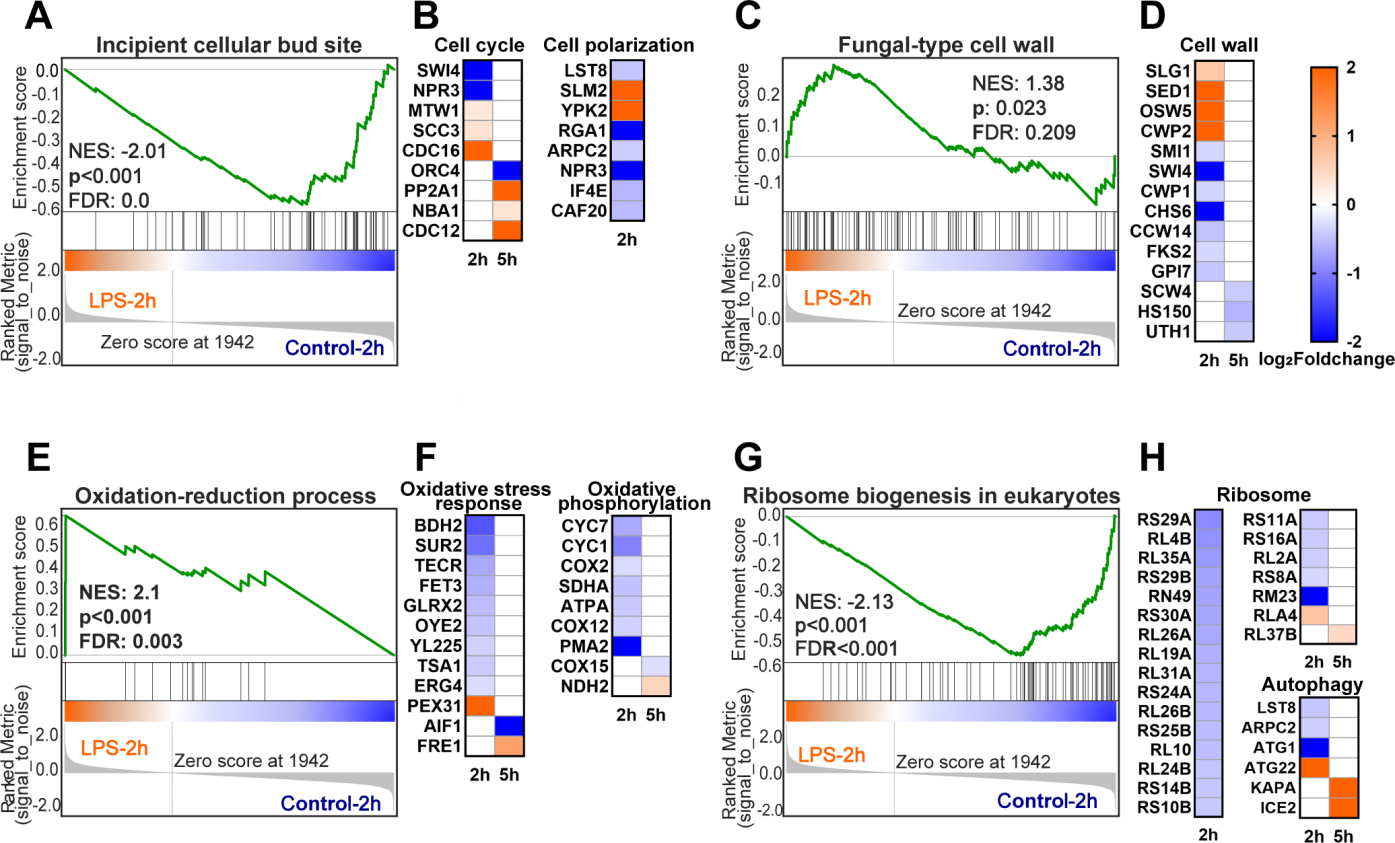
Molecular mechanistic insights on the time-resolved cell response to LPS exposure. (**A**) GO term “incipient cellular bud site” in transcriptomic GSEA results. (**B**) The proteins related to cell cycle and cell polarization. (**C**) GO term “fungal-type cell wall” in transcriptomic GSEA results. (**D**) Cell wall-associated proteins. (**E**) GO term “oxidation-reduction process” in transcriptomic GSEA results. (**F**) Proteins associated with oxidative stress response and oxidative phosphorylation. (**G**) KEGG term “ribosome biogenesis in eukaryotes” in transcriptomic GSEA results. (**H**) The proteins associated with ribosome and autophagy. In heatmaps, each square represents a protein, with blue indicating downregulation and orange indicating upregulation. These heatmaps only show DEPs with *P* values < 0.05 and FC >1.2 or FC <0.83.

The proteins involved in cell growth and division are regulated at both the transcriptomic and proteomic levels (Fig. 4A and B). This phenomenon is most prominent in yeast samples exposed to LPS for 2 hours. The proteins related to the cell cycle include transcription factors SWI4, cohesin subunit SCC3, anaphase-promoting complex subunit CDC16, and kinetochore-associated protein MTW1 (45, 46). Analysis of transcriptomic and proteomic data showed that exposure to LPS for 2 hours resulted in overall downregulation of the cell cycle and inhibition of cell mitosis. After 5 hours of exposure, cell cycle-related DEPs included origin recognition complex subunit 4 (ORC4) and catalytic subunit of protein phosphatase 2A (PP2A1). This means that prolonged exposure to LPS will continuously affect the cell cycle of yeast. Synergistic changes were observed between cell germination rate and cell cycle. GSEA showed that the gene expression was significantly enriched in the GO terms “incipient cellular bud site,” “cellular bud tip,” and “cellular bud neck” in the control group compared to the LPS-treated group (Fig. 4A; Fig. S9). After 2 hours of exposure to LPS, the TORC2 signaling pathway and cell polarization-related proteins related to budding were regulated (Fig. 4B). Rapamycin complex 2 (TORC2) is an evolutionarily conserved regulatory molecule for eukaryotic cell growth, closely related to actin polarization in yeast cells, and regulates cell spatial growth (47–49). It is worth noting that establishing cell polarity in the correct direction is crucial for cell budding. Rho type GTPase-activating protein 1 (RGA1) prevents budding within the division site by regulating the cell polarity axis. The downregulation of RGA1 results in polarized cells being unable to select suitable germination sites (50), leading to a decrease in the germination efficiency. After 5 hours of exposure to LPS, proteins CDC12 and NBA1 related to cell budding were upregulated. NBA1 can synergistically interact with RGA1 to avoid cell repolarization at previous division sites, thereby facilitating cell budding (51). The evidence presented above indicates that LPS exposure influences yeast cell budding by regulating the cell cycle and the sites of polarized cell budding. This effect is observed to be persistent, although it tends to be mitigated after 5 hours of exposure.

Exposure to LPS can affect the integrity pathway of yeast cell walls and cause cell wall remodeling. Transcriptomic and proteomic data provide evidence that processes related to the cell wall are greatly impacted (Fig. 4C and D). GSEA showed that the gene expression was significantly enriched in the GO term “fungal-type cell wall” in the LPS-treated group compared to the control group. The proteomic data exhibit the regulation of multiple proteins implicated in the cell wall changes (Fig. 4D). Changes in cell wall-related proteins can be observed after 2 hours of exposure, and this effect persists for up to 5 hours. The SLG (WSC1) protein operates as a sensor enhancing cell wall adaptability to external stimuli by conveying its upregulation through the cell wall integrity (CWI) signaling pathway (52). Proteins associated with glucan and chitin synthesis downstream of the CWI pathway are downregulated (53). This implies that glucan and chitin synthesis are hindered and might result in cell wall loosening, leading to the secretion of vesicles. After 2 hours of exposure, the structural protein of the cell wall, the glycosylphosphatidylinositol (GPI) anchor protein, was also regulated (54). Furthermore, over the exposure time, the DEPs associated with cell walls decreased after 5 hours. Similarly, low expression of glucosidases UTH1 was found to regulate the biogenesis of yeast cell walls, making them more resistant (55). Thus, these findings support the idea that cell wall remodeling is the primary response mechanism for yeast to LPS stress and suggests adaptive regulation over time.

In response to LPS exposure, yeast cells engage in a series of defensive processes, including oxidative stress and detoxification responses (Fig. 4E and F). Additionally, the oxidative phosphorylation process has been identified as a potential source of ROS. After exposure to LPS, the redox process of yeast cells changes, and multiple redox enzymes related to ROS are regulated (Fig. 4F). The main method used by cells to counteract the accumulation of ROS is to chemically reduce them using the thiol group (- SH) of the antioxidant peptide glutathione (56). The downregulated antioxidant proteins in the 2-hour sample include glutathione 2 (Grx2) (57), thioredoxin peroxidase TSA1 (58), and NADPH dehydrogenase 2 (OYE2) (59). In addition, the upregulated peroxisome-integrated membrane protein PEX31 is a negative regulatory factor for peroxisomes (60). The regulation of antioxidant proteins and PEX31 indicates a decrease in the ability of yeast cells to resist ROS after 2 hours of exposure to LPS. After 5 hours of exposure to LPS, it was observed that apoptosis-inducing factor 1 (AIF1) and ferric/complementary reductase 1 (FRE1) were regulated. This regulation can improve the tolerance of cells to oxidative stress and delay cell death (61–63). Therefore, transcriptomic and proteomic analyses indicate that exposure to LPS triggers sustained oxidative stress in yeast cells. And the oxidative stress-tolerant proteins were observed after 5 hours of exposure, indicating that yeast cells tend to detoxify and adapt. The respiratory chain during mitochondrial oxidative phosphorylation is the main source of intracellular ROS (64, 65). The downregulation of several proteins, which make up mitochondrial complex IV, may lead to incomplete catalysis of oxygen and the production of ROS at complexes I and III (66). Downregulation of cytochrome C proteins may restrict their function, leading to incomplete oxidation and excessive production of superoxide anions (67). It has been reported that oxidative stress and ROS can alter the activity of ethanol dehydrogenase, contributing to the metabolism of pyruvate to produce acetic acid. This may also serve as an endogenous sink for ROS (68). It is worth noting that the ATP synthase subunit alpha (ATPA) responsible for the synthesis of ATP at the end of the oxidative phosphorylation pathway is downregulated, indicating a weakening of energy metabolism. The regulation of energy metabolism can seriously affect the physiological processes of cells, leading to autophagy and even programmed cell death. In the samples exposed to LPS for 2 hours, it was found that “ribosome biogenesis in eukaryotes” was downregulated at the gene expression (Fig. 4G), and 21 ribosomal proteins were also downregulated (Fig. 4H). This finding is consistent with that of a previous study that reported a similar downregulation of ribosome biogenesis-related genes (26). In addition, ATG22, a protein associated with autophagy, was upregulated (Fig. 4H), which contributes to the exocytosis of amino acids during autophagosomal catabolism, thus altering the levels of intracellular metabolic substrates (69). Affecting amino acids, as discussed above, could also affect the production of VOC species by the cell. In conclusion, the inhibition of mitochondrial oxidative phosphorylation leads to decreased ROS production and energy metabolism, which further affects the activity and substrate of cell metabolism. VOCs as small-molecule metabolites may reflect the level of oxidative stress and the state of energy metabolism directly related to their metabolism.

### VOCs as cell stress response markers

**Fig 5:**
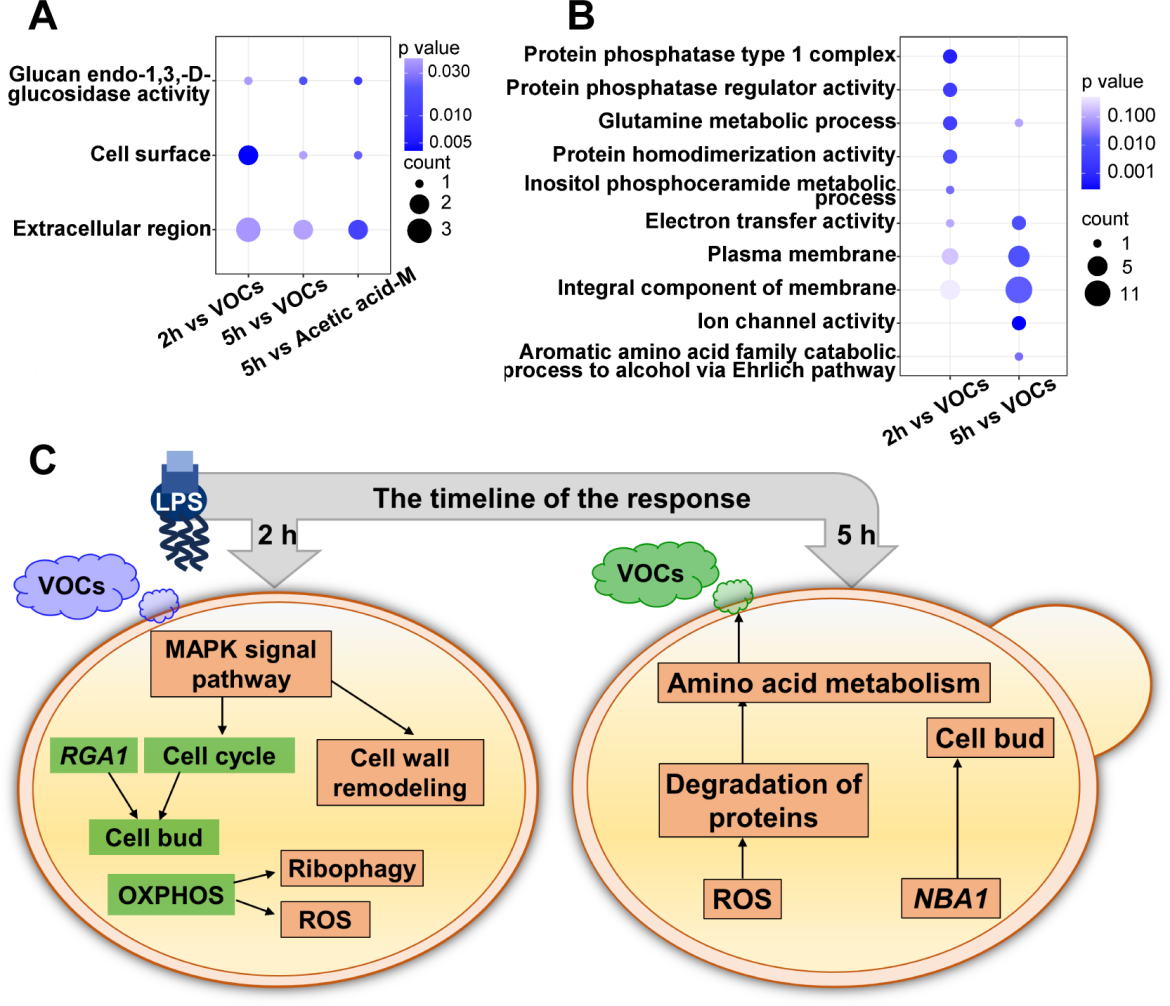
VOCs can serve as markers for the different states of cellular stress. (**A**) Protein pathways significantly correlated with VOC expression by GO enrichment. (**B**) Gene pathways significantly correlated with VOC expression obtained by GO enrichment. (**C**) Molecular mechanisms underlying the release of characteristic VOCs during the stress and adaptive response phases of yeast exposure to LPS. Downregulated biological pathways and proteins are labeled using green rectangles. Upregulated biological pathways are labeled with orange rectangles.

Yeast cells have different characteristic VOCs and gene expression patterns at different periods after LPS exposure. Correlation analysis can provide additional insights into their relationship. The statistical results have indicated a significant correlation (*P* value < 0.05) solely between the level of acetic acid-D and the expression of gene or protein in the 2-hour samples (Fig. S10). In total, the levels of the four VOCs were significantly correlated with the expression of 18 proteins and 35 genes in the 5-hour sample (*P* value < 0.05) (Fig. S11). To explore the molecular mechanism of VOCs as an early warning of adverse cellular states, proteins or genes with significant correlations between VOCs were further analyzed by GO enrichment. The acetic acid-D was upregulated in both the 2-hour and 5-hour samples. Thus, separate analyses of proteins significantly correlated with acetic acid-D in the 2-hour sample, proteins significantly correlated with acetic acid-D in the 5-hour sample, and proteins significantly correlated with all VOCs in the 5-hour sample revealed an intersecting pathway between the three (Fig. 5A). This suggests that increased levels of acetic acid-D may represent the regulation of the pathways “cell surface,” “glucan endo-1,3-beta-D-glucosidase activity,” “extracellular region,” and “cell surface” at the protein level. These pathways align with known cell wall remodeling during stress. However, acetic acid is the high oxidation state endpoint of multiple metabolic pathways, and the specific mechanism responsible for its metabolic upregulation is not clear. In contrast, we did not reach similar conclusions in our analysis of genes significantly associated with acetic acid-D. The analysis of all VOCs significantly related genes in the 2-hour sample revealed that the pathways related to protein phosphorylation and homodimerization were enriched. Among the identified genes, the two regulatory subunits of the Glc7p type-1 protein phosphatase gene exhibited a notable decrease in expression. This finding is associated with the regulation of glucose metabolism during the cellular stress phase (70). The analysis of all VOCs significantly related genes in the 5-hour sample revealed that pathways related to the plasma membrane and metabolic status were significantly enriched. The genes encoding heat shock proteins and ion channel proteins were significantly downregulated, which contributed to prolonged cellular lifespan by maintaining intracellular homeostasis (71). It is noteworthy that all VOCs other than acetic acid-D in the 5-hour samples were higher alcohols/aldehydes. This seems to correspond to the results of the gene enrichment analysis (Fig. 5B). The pathway “aromatic amino acid family catabolic process to alcohol via Ehrlich pathway” is attributed to the upregulation of the *PDC6* gene encoding decarboxylase, which catalyzes the decarboxylation of α-keto acids to form aldehydes during amino acid catabolism. Aldehydes are further oxidized or reduced to acids or alcohols. Although there was an upregulation trend of gene *PDC6* in the corresponding samples after 5 hours of LPS exposure, the release of propionaldehyde-M was downregulated. This phenomenon may be due to limitations in the detection technology and the quick Ehrlich reaction process. The decrease in propionaldehyde release is primarily due to the accelerated rate of 1-propanol production as compared to the control group following LPS exposure. The alterations observed in 1-propanol-D are significantly and negatively associated with those in propionaldehyde (r = −0.82, *P* value < 0.0001) (Fig. S12). The concentration of 1-propanol-D released correlates positively with the expression level of the *PDC6* gene (r = 0.76, *P* value = 0.079) (Fig. S12). Additionally, although alcohol dehydrogenase or aldehyde dehydrogenase is required in addition to the decarboxylase encoded by *PDC6* in the metabolism of higher alcohols, we observed a positive correlation between the *PDC6* gene and other VOCs. The expression level of the *PDC6* gene is positively correlated with the release concentrations of 1-butanol-M (r = 0.59, *P* value = 0.22), 3-methylbutanol-D (r = 0.77, *P* value = 0.073), and acetic acid-D (r = 0.87, *P* value = 0.025) (Fig. S12). In summary, the aldehydes catalytically generated by *PDC6* may undergo rapid oxidation or reduction to acids or alcohols, which is responsible for the characteristic VOC profile of yeast during the *in vivo* adaptation phase. The specific regulatory mechanism leading to the changes in the level of VOC release from yeast is unknown and requires further investigation. Nonetheless, correlation analyses at least provide us with clues about the possible associations of protein/gene regulation with the observed VOC species.

The potential mechanisms behind the changes in VOC release may involve the modulation of metabolic substrates and oxidative stress levels through stress-responsive signaling networks. The VOCs released by yeast cells are considered to be generated through various metabolic activities. Among these, the VOCs produced by the primary metabolic pathway are usually considered waste from the detoxification process, while those produced by secondary metabolism are considered a defense or communication mechanism (72). Based on our research findings and previous studies, we believe that the following mechanisms for the VOC production by *S. cerevisiae* apply in response to toxic stress and adaptive pathways, as shown in Fig. 5C. The yeast damage caused by LPS exposure is mainly due to the production of ROS, which leads to autophagy of ribosomes and changes in intracellular nutrient levels. In addition, this phase is accompanied by a stress response to growth inhibition and cell wall remodeling processes. According to reports, ethyl acetate is a VOC marker released by yeast cells in response to H_2_O_2_ stimulation. Therefore, acetic acid and its ester derivatives may serve as biomarkers for oxidative stress in cells (23). Yeast uses amino acids released from autophagy as metabolic substrates in the adaptive pathway and converts pyruvate into higher alcohols/aldehydes through the Ehrlich pathway via secondary metabolism (73). Therefore, higher alcohols/aldehydes may serve as markers for cell adaptation to LPS exposure.

This study investigated the changes in VOC release over time in cells stimulated by LPS and integrated multi-omics analyses to explain the regulatory network behind it. Here, we detected changes in VOC composition released from yeast cells after 2 and 5 hours of exposure compared to the control. Integrating multi-omics data, different sets of VOC species over time can be used to describe a particular endpoint of the cellular response to external stimuli and thus serve as indicators for the life processes. Notably, acetic acid can serve as a signal for yeast cells to respond to LPS stress, whereas higher alcohols/aldehydes can serve as markers of adaptive processes in yeast cells. Moreover, the key advantage of VOC monitoring lies in its real-time feasibility, unlike conventional cellular assays. Although the mechanistic details require further exploration, this work provided time-resolved measurements of relative changes in VOCs released by the yeast cells upon LPS exposure. *S. cerevisiae* has genomic homology with humans, and the results could provide hints for understanding the cellular responses of human cells upon exposure. This study underscores the potential of VOC-based “surveillance” for biological health monitoring.

## MATERIALS AND METHODS

### Strains, culture conditions, and LPS treatment

The *S. cerevisiae* used in this work is strain S288c (ATCC 204508), which is one of the most commonly used model strains in the laboratory for protein and human disease studies. All experiments were performed in complete synthetic (SC) media to facilitate monitoring and standardization. The formula for SC medium includes the following: 2% (wt/vol) glucose (Sinopharm Chemical Reagent, China), 0.67% (wt/vol) yeast nitrogen base (Sigma, USA), and 0.079% (wt/vol) complete supplement mixture (MP Biomedicals, USA). The yeast cells were pre-grown at 30°C on an orbital shaker (200 rpm). A yeast inoculum grown in the logarithmic phase was used for the experiment. The stress element used in the experiment was LPS from *Escherichia coli* O111:B4 (Sigma, USA, L4391). The yeast inoculum was counted using a hemocytometer and diluted to 5 × 10^7^ cells/mL for the following LPS exposure. The untreated cells were used as controls.

### Yeast growth and survival assays

Yeast cell viability under LPS exposure was assessed using growth curve analysis, plate culture, and spot assays. Cells were incubated in the SC medium in the absence or presence of LPS (5 µg/mL). For growth curve measurement, we used a hemocytometer to measure the cell concentration of yeast culture every hour. For the plate culture method and the spot test method, yeast cultures were tested at 2- and 5 hour post-exposure. After incubation, the yeast cultures were serially diluted at 10 x and spread or spotted onto SC agar plates. Plates were incubated at 30°C for 48 hours, photographed, and colony-forming units (CFU) quantified. Yeast cell viability without LPS treatment was considered to be 100%.

### Cellular localization detection of LPS

This study used fluorescein isothiocyanate (FITC)-labeled LPS (F8666, Sigma, USA) instead of LPS for exposure experiments with yeast cells (exposure concentration of 10 µg/mL) in order to track the interaction between LPS and yeast cells. Cells treated solely with an equivalent molar amount of the FITC fluorophore (F8070, Solarbio, China) as present in FITC-LPS, or with potassium phosphate buffer (PBS, pH 7.4) alone, served as the control groups. For samples designated as exposure time “0 hour,” they refer to the time when cells were first exposed to FITC-LPS or FITC or PBS and then immediately followed by washing twice with ice-cold PBS. For the convenience of observation, the cell membrane damage probe propidium iodide (PI) (CP9161, Coolaber, China) was employed to assist in detection. Yeast cells exposed to FITC-LPS or FITC or PBS were collected, washed twice with PBS (centrifuged at 1,000 × g for 5 minutes), and resuspended in PBS. PI (working concentration of 20 µg/mL) was added to the sample, which was then incubated in the dark at 30°C for 30 minutes. Finally, the sample was washed twice with PBS (centrifuged at 1,000 × g for 5 minutes). These samples were subsequently used for observation with an AX upright laser scanning confocal microscope (AX, Nikon, Japan) and for analysis with a flow cytometer (FACSVerse, Becton Dickinson, USA).

For laser scanning confocal microscopy, FITC-LPS was detected using an excitation wavelength of 488 nm and an emission wavelength of 500–550 nm. PI was detected using an excitation wavelength of 561 nm and an emission wavelength of 571–625 nm. The cell localization of FITC-LPS was determined through Z-stack imaging, with optical sections taken at a step size of 0.6 µm. Imaging was acquired with a 60 x oil immersion objective, and the images were processed using NIS Elements Viewer ver.5.22 software. In flow cytometry analysis, FITC-LPS and PI were detected using the FITC channel (495–559 nm emission) and the PE channel (544–628 nm emission) at an excitation wavelength of 488 nm. Fluorescence sensitivity thresholds: FITC ≤100 MESF and PE ≤25 MESF. Single-stained samples are utilized as transition samples in flow cytometry to adjust voltage compensation and prevent crosstalk between the two channels. Flow cytometry data for 20,000 cells were obtained using the FACSVerse, and data analysis was performed with FlowJo ver.10.8.1 software.

### Cellular ROS assay

Intracellular reactive oxygen species (ROS) were detected and visualized after LPS treatment. Yeast cells were collected, centrifuged at 1,000 × g for 5 min, washed three times with PBS, and then resuspended in PBS. Intracellular ROS were determined by staining with 2′,7′-dichlorofluorescein diacetate (DCFH-DA) (Abcam, UK). DCFH-DA was incubated using a working concentration of 2 µmol/L for 30 min at 30°C. After centrifugation, the stained cells were collected and washed in PBS three times and finally resuspended in PBS. ROS was quantified by SPECTROMAX II microplate reader (Molecular device, USA) under 485 nm excitation and 535 nm emission. The stained cells were observed by a BX63 fluorescence microscope (Olympus, Japan).

### Preparation of omics samples

Samples were collected at 2 and 5 h after LPS exposure of yeast cells based on the results of extracellular metabolism experiments. The control groups without LPS treatment were also included. The number of biological replicates for transcriptomic, proteomic, and metabolomic analyses was 3, 3, and 6, respectively. Except for metabolomic samples, the samples were collected by centrifugation at 1,000 × g at 4°C for 5 min and washed with PBS. Yeast cells were snap-frozen with liquid nitrogen and stored at −80°C for analysis.

### Transcriptomic analysis

Total RNA from yeast cells was extracted using TRIzol reagent (Invitrogen, CA, USA) according to the manufacturer’s instructions. Transcriptome library construction and sequencing were performed by OE Biotechnology Co., LTD. (Shanghai, China). The quality of RNA was measured using a NanoDrop 2000 spectrophotometer (Thermo Fisher Scientific, USA) and an Agilent 2100 Bioanalyzer (Agilent Technologies, Santa Clara, CA, USA) before sequencing (RNA quantity >1 µg, relative intensity noise (RIN) ≥7.0, 28S/18S ≥ 1.0). Transcriptome libraries were constructed using the VAHTS Universal V5 RNA-seq Library Prep kit (Illumina, San Diego, CA, USA). Then, the libraries were sequenced on the Illumina Novaseq 6000 sequencing platform, and 150-bp paired-end reads were generated. For clean data, all raw data (raw reads) were cleaned, and low-quality reads were removed.

### Metabolomic analysis

Yeast cell exposure to LPS was performed in a Drechsel gas-washing bottle. During the detection process, the Drechsel gas-washing bottle was placed in a constant temperature water bath at 30°C to maintain the activity of yeast cells. When VOCs were not detected, the bottle was sealed with a sterile ventilated sealing film and incubated at 30°C on the orbital shaker. Yeast extracellular metabolites, VOCs, were analyzed by a BreathSpec GC-IMS (G.A.S., Dortmund, Germany). The instrument consists of a gas chromatograph (GC) and an ion mobility spectrometer (IMS). For online analysis of VOCs, zero air consisting of only O_2_ and N_2_ (2:8) (Beijing AP BAIF Gases Industry Co., Ltd.) was connected to the inlet tube of the bottle and used as a carrier gas for the VOCs. The carrier gas flow rate used for sample determination was 0.3 L/min, and after the gas flow stabilized for 1 minute, GC-IMS was used to measure VOCs. The GC-IMS inlet port was connected to the outlet port of the bottle for the analysis of VOCs (Fig. S13). The GC-IMS injection port was wrapped with a 45°C insulation sleeve to prevent water vapor condensation and sample loss. This direct injection method does not require sample enrichment and collection and can quickly detect VOCs. We continuously monitored the characteristics of VOCs emitted by *S. cerevisiae* after LPS exposure at 1-hour intervals. Two-dimensional data were obtained from GC-IMS detection, namely, the retention index (RI) of the molecule in GC and the drift time (DT) in IMS. VOCs were identified by VOCal software and GC-IMS library (V0.1.1, G.A.S.) based on DT and RI values. The relative amounts of VOCs were determined from the software peak areas. For undetermined VOC species that did not match the corresponding pair of values in the GC-IMS library, they were labeled with the corresponding area #s.

For intracellular metabolites, two independent analytical methods were used, including liquid chromatography coupled with mass spectrometry (LC-MS) and gas chromatography coupled with mass spectrometry (GC-MS). After cell fragmentation, derivatization reactions were performed on the cell contents and detected. For intracellular metabolites, yeast cells were broken up by sonication in an ice-water bath using pre-cooled 1 mL of methanol/water (V: V = 1:1) and 200 µL of chloroform as the extractant. After adding 40 µL of the internal standard (L-2-chlorophenylalanine dissolved in methanol, 0.1 mg/mL), the extraction was continued for 20 min in an ice-water bath. Samples were centrifuged at 12,000 rpm for 10 min, and the supernatant was stored for dual-instrument analysis. For LC-MS, the supernatant was filtered through a 0.22 µm filter and transferred to the LC injection vial for instrumental analysis. For GC-MS, the supernatant was dried under vacuum at room temperature and subsequently mixed with 80 µL of methoxyamine hydrochloride pyridine solution (15 mg/mL) and shaken at 37°C for 60 min for an oxime reaction. To obtain this mixture, 50 µL BSTFA and 20 µL hexane were added, and the derivatization reaction was carried out at 70°C for 60 min. After the samples were cooled to room temperature, they were subjected to instrumental analysis. The analysis of intracellular metabolites was performed by Luming Biotech Co., Ltd. (Shanghai, China). The analytical instruments included GC-MS 8890-5977B (Agilent Technologies Inc., CA, USA), ACQUITY UPLC I-Class system coupled with VION IMS QTOF Mass spectrometer (Waters Corporation, Milford, USA).

### Proteomic analysis

Proteins were extracted from three biological replicates of each set of samples using the ultrasonic fragmentation method. Protein concentrations were determined by BCA assay (Thermo Fisher Scientific, USA) and were trypsinized. The desalted peptides were separated using liquid chromatography-tandem mass spectrometry (LC-MS/MS) (Thermo Fisher Scientific, USA). Liquid chromatography was coupled online with a hybrid TIMS quadrupole TOF mass spectrometer (Bruker, Germany) for data acquisition. Proteomic sequencing was entrusted to Shanghai Luming Biotechnology Co. (Shanghai, China). According to the measured protein concentration, an equal amount of protein was extracted from each sample, and samples from different groups were diluted to the same concentration and volume. An appropriate volume of 25 mM DTT was added to the protein solution mentioned above to achieve a final DTT concentration of approximately 5 mM and incubated at 55°C for 30 minutes. Then, an appropriate volume of iodoacetamide was added to the solution to achieve a final concentration of approximately 10 mM and left at room temperature for 15 minutes. The protein was then precipitated by adding six times the volume of precooled acetone to the system mentioned above and left at 20°C overnight. Subsequently, the sample was centrifuged at 8,000 × *g* and 4°C for 10 minutes, and the precipitate was collected. Based on the amount of protein, an appropriate volume of enzyme digestion dilution solution was added (protein: enzyme = 50: 1, wt/wt; 100 µg protein with 2 µg enzyme), and the protein precipitate was dissolved again. The digestion was carried out at 37°C for 12 hours, followed by lyophilization.

All analyses were performed by a Tims TOF Pro mass spectrometer (Bruker, Germany) equipped with an Easyspray source (Thermo Fisher Scientific, USA). The sample was loaded onto a capillary trapping column (100 µm × 2 cm, RP-C18, Thermo Fisher Scientific) and separated on an analytical column (75 cm ×18 µm, RP-C1200, Thermo Fisher Scientific) using the EASY-nLC 15 system (Thermo Fisher Scientific). Gradient elution of 3% to 27% to 46% to 100% was performed within 90 minutes using a mobile phase of ACN: H_2_O: FA (80:19.9:0.1). Ion mobility is set from 0.6 to 1.6 Vs/cm^2,^ and the collision energy ranges from 20 to 59 eV. Full MS scans were acquired in the mass range of 100–1,700 *m/z*. MS/MS spectra were searched using the MaxQuant against the UniProt-*Saccharomyces cerevisiae* database. Search database specific parameters are set as follows: fixed modifications (carbamidomethyl (C)), variable modification (oxidation (M) and acetyl (protein N-term)), digestion: trypsin; first search peptide tolerance (20 ppm), main search peptide tolerance (10 ppm), and missed cleavage.

### Statistical analysis

All data represented by SEM ±mean represent at least three independent experiments. *t*-tests for cell growth and ROS were performed using Prism (v8.0, GraphPad, USA). DEGs in yeast cells induced by LPS exposure were analyzed using DESeq2 (V1.24.0), determined based on *P* value < 0.05 and fold change (FC) >1.5 or FC <0.67. We used italics to denote genes in the text and pictures. The extracellular metabolite and intracellular metabolite data were analyzed using SIMCA software (V14.1, Umetricus, Sweden) of principal component analysis (PCA), partial least squares discriminant analysis (PLS-DA), and orthogonal partial least squares discriminant analysis (OPLS-DA) models. The PCA model was applied to evaluate overall differences among extracellular metabolite groups. Standardized data were used to generate a covariance matrix for PCA. To complement the PCA results, multivariate analysis of variance (MANOVA) was performed on the principal components (PC1 and PC2). The PLS-DA model and OPLS-DA model helped analyze the differences in metabolite expression. Moreover, metabolites with VIP >1.0 and *P* value < 0.05 were considered DEMs. DEPs in yeast cells induced by LPS exposure were determined based on *P* value < 0.05 and FC >1.2 or FC <0.83.

Gene Ontology (GO) annotation and pathway analysis are used to explore the role of DEGs and DEPs. In short, GO annotation is the discovery of gene regulatory networks based on hierarchical categories, based on the molecular functions, biological processes, and cellular components of differentially expressed genes or proteins. We performed pathway analysis based on Kyoto Encyclopedia of Genes and Genomes (KEGG) to investigate important pathways enriched with differentially expressed genes. Gene expression pattern analysis is performed using the short-time sequence expression miner (STEM) software (74), which allows researchers to identify significant time course profiles and genes associated with these profiles and to compare the behavior of these genes under different conditions. All data were filtered by mathematical models to remove data with insignificant differences in time gradient expression. The false discovery rate (FDR) method was used to correct the *P* value, and modules with *P* values less than 0.05 were selected as significant. Gene set enrichment analysis (GSEA) was performed to analyze the enrichment pathways generated from the two DEG sets described above (75), with a cutoff of the absolute value of the normalized enrichment score >1, FDR < 0.25, and *P* value < 0.05.

### Quality assurance/quality control (QA/QC)

All experiments were performed in at least three biological replicates. For all experiments, the yeast culture was purified/cleaned before the experiment by replacing the supernatant after centrifugation with the fresh SC medium. The purification processes were repeated three times to eliminate the metabolites generated during the yeast cultivation. For the analysis of the extracellular metabolites, the stability of GC-IMS was verified by parallel injection of a mixture of 6 ketones (2-butanone, 2-pentanone, 2-hexanone, 2-heptanone, and 2-nonanone) as quality QC samples. For intracellular metabolite analysis, all samples were mixed into QC samples and tested in the same batch. Ion peaks with relative standard deviation >30% in the QC sample group were removed.

## Data Availability

The transcriptomic data generated during this study have been deposited at the National Genomics Data Center (NGDC) (https://ngdc.cncb.ac.cn/gsa) under accession number CRA013968. The original proteome data have been deposited in the ProteomeXchange Consortium via the iProX partner repository with the data set identifier PXD047920.

## SUPPLEMENTAL MATERIAL

Supplemental material aem.00785-25-s0001.docx Figures S1 to S13; Tables S1 to S3.

ASM does not own the copyrights to Supplemental Material that may be linked to, or accessed through, an article. The authors have granted ASM a non-exclusive, world-wide license to publish the Supplemental Material files. Please contact the corresponding author directly for reuse.
