# Supplementary material for: Time-resolved monitoring of yeast responses to lipopolysaccharide exposure by cell-released volatile organic compounds
Source: Appl Environ Microbiol. 2025 Aug 6;91(9):e00785-25. doi: 10.1128/aem.00785-25 (PMC12442379; doi:10.1128/aem.00785-25)
Supplement: Supplemental material — Figures S1 to S13; Tables S1 to S3. [file aem.00785-25-s0001.docx]

**Supplemental Material**

**for**

**Time-resolved Monitoring of Yeast Responses to Lipopolysaccharide Exposure by Cell-Released Volatile Organic Compounds**

Huaying Liu and Maosheng Yao*

State Key Joint Laboratory of Environmental Simulation and Pollution Control, College of Environmental Sciences and Engineering, Peking University, Beijing 100871, China

***Corresponding Author**:

Maosheng Yao, PhD

Boya Distinguished Professor

E-mail: yao@pku.edu.cn, Tel: +86 010 62767282

**Content of Supporting Information**

[Supplementary Figures 1](#_Toc169027174)

[Supplementary Tables 14](#_Toc169027175)

## Supplementary Figures


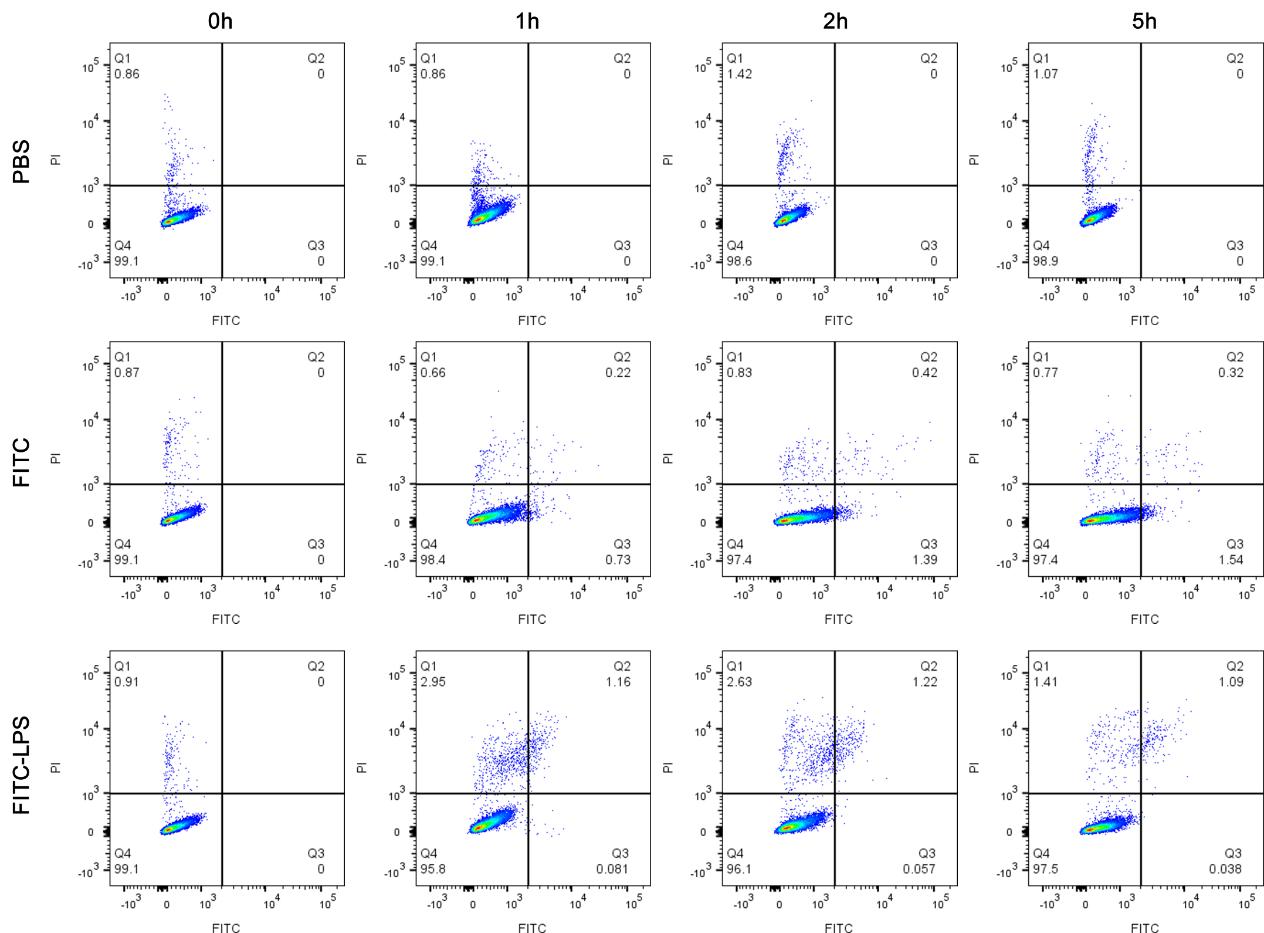


**Fig. S1 Flow cytometry analysis of yeast exposed to FITC-LPS.** Membrane-damaged cells not bound to FITC-LPS or FITC are represented in Q1. Membrane-damaged cells bound to FITC-LPS or FITC are represented in Q2. Intact cells bound to FITC-LPS or FITC are represented in Q3. Intact cells not bound to FITC-LPS or FITC are represented in Q4. Fluorescence sensitivity thresholds: FITC ≤100 MESF and PE ≤25 MESF.


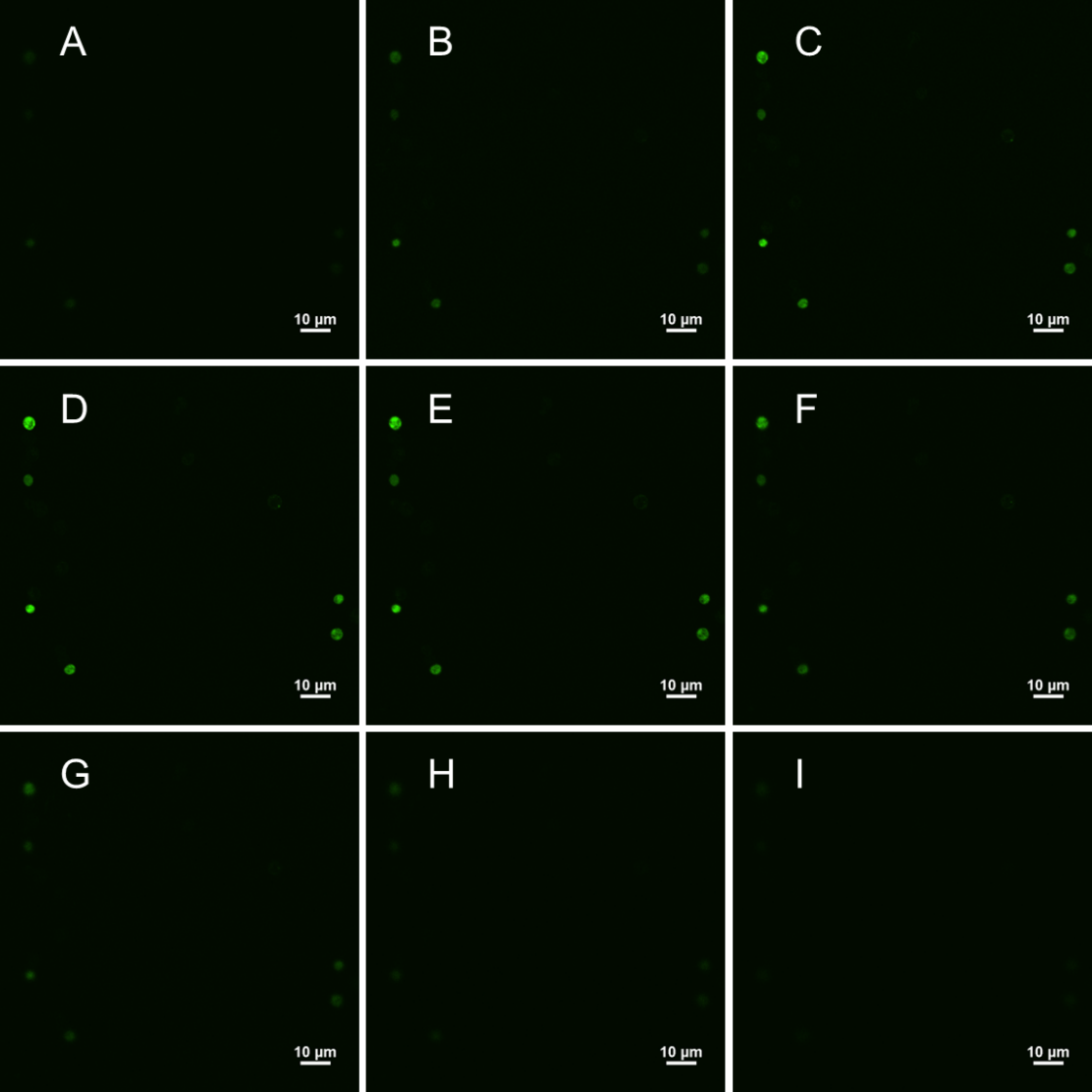


**Fig. S2 Laser scanning confocal microscopy analysis of FITC-LPS cell localization.** (A-I) Z-stack images show consecutive optical sections (each of 0.6 μm thickness).


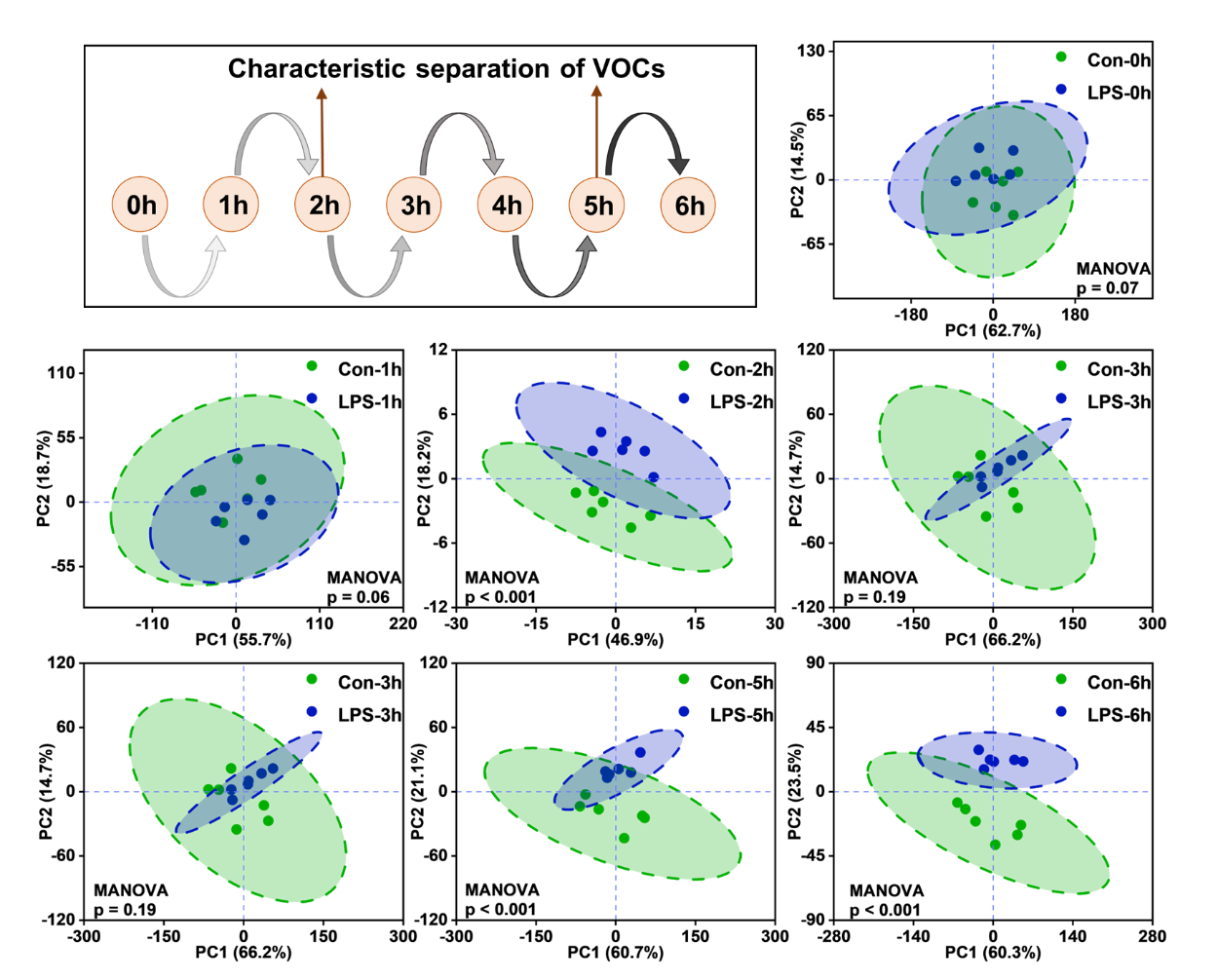


**Fig. S3 The principal component analysis of hourly VOCs.** The ellipses in the fig. represent 95% confidence intervals. P values are derived from multivariate analysis of variance (MANOVA).


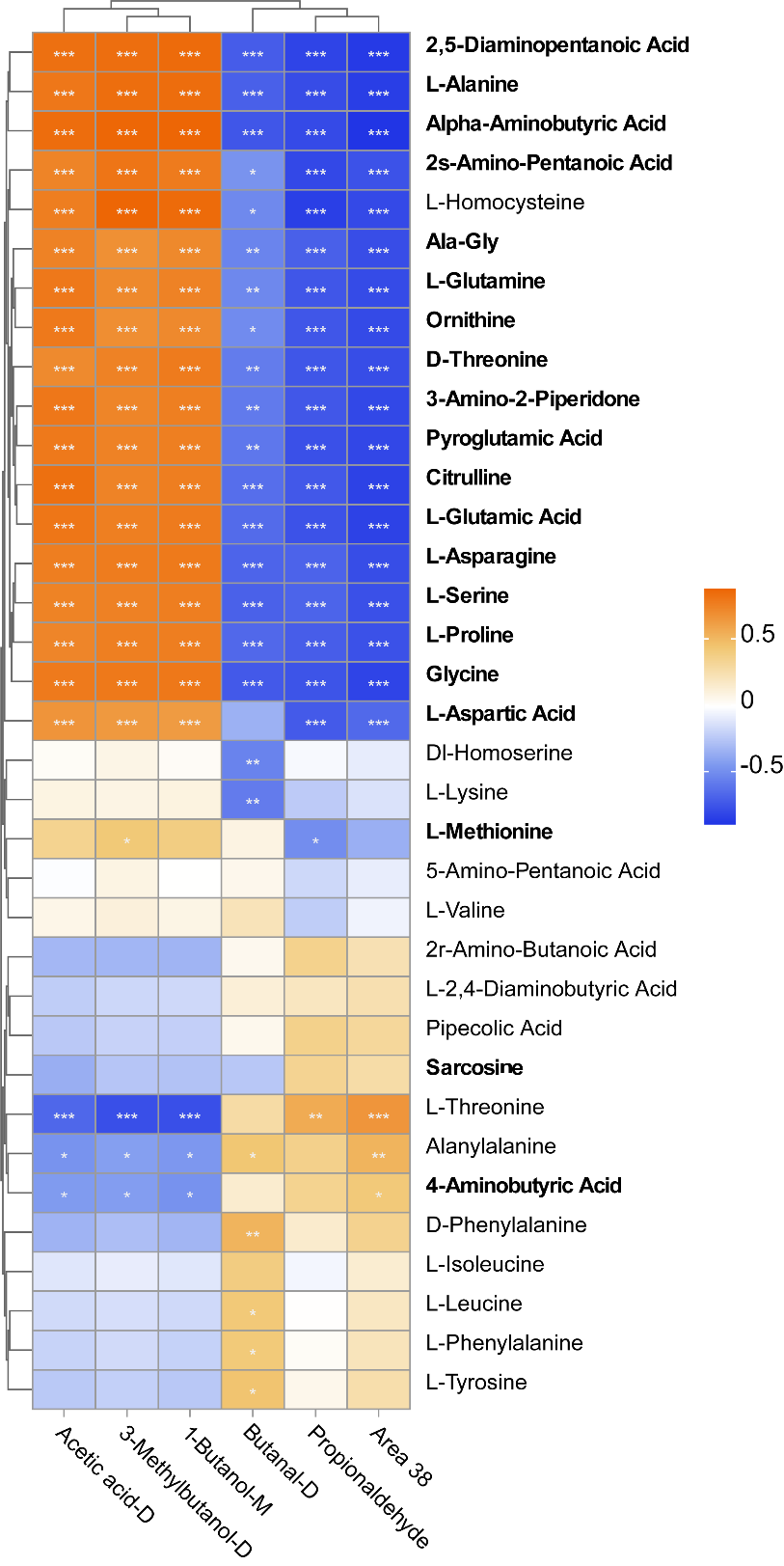


**Fig. S4** **Pearson correlation analysis of VOCs signal values with intracellular amino acid signal value.** Data include all 24 samples at 2 hours and 5 hours. *, **, *** represent significant correlation at 0.05,0.01,0.001 level respectively.


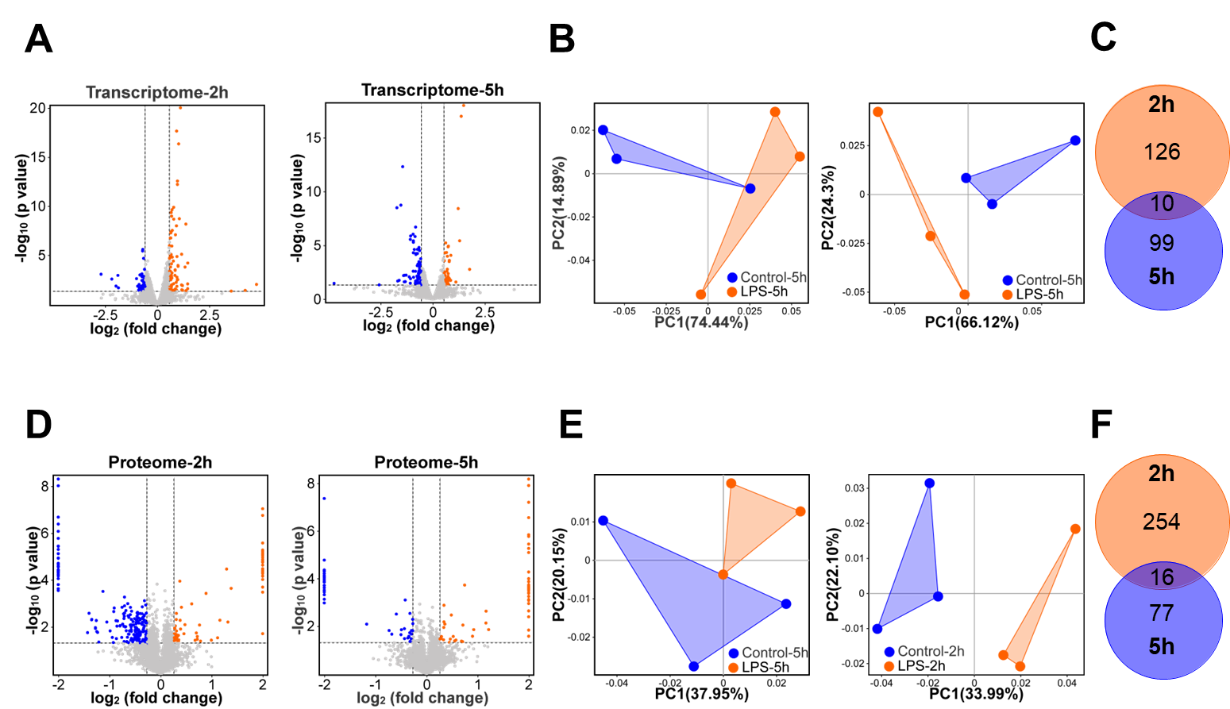


**Fig. S5** **Transcriptomic and proteomic profiles.** (A) DEGs that were upregulated or downregulated after 2 or 5 hours of LPS exposure. (B) Principal component analysis of transcriptomic data after 2 and 5 hours of LPS exposure. (C) Venn diagram of DEGs under LPS exposure vs control conditions after 2 and 5 hours. (D) DEPs that were upregulated or downregulated after 2 or 5 hours of LPS exposure. (E) Principal component analysis of proteomic data after 2 and 5 hours of LPS exposure. (F) Venn diagram of DEPs under LPS exposure vs control conditions after 2 and 5 hours.


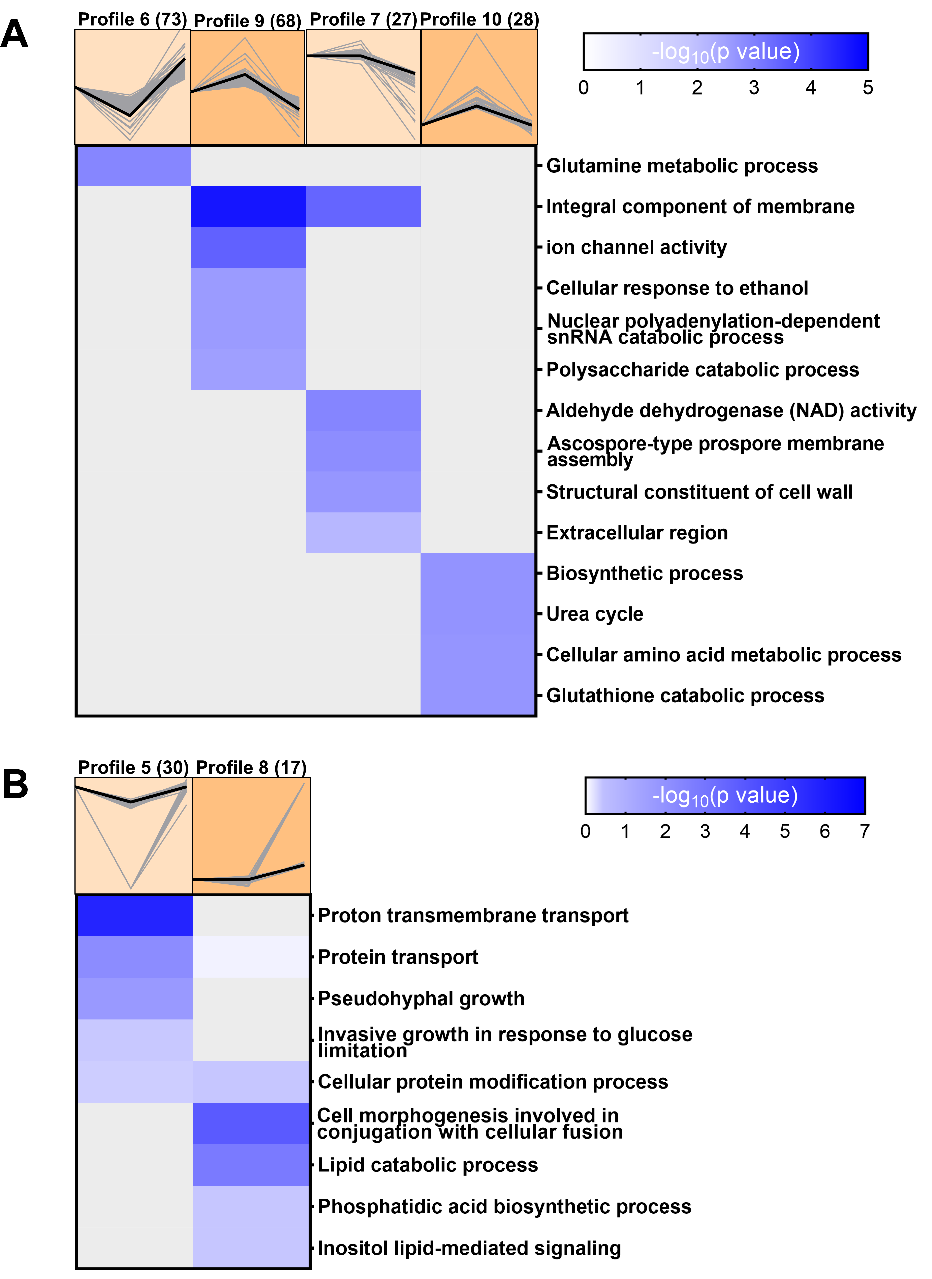


**Fig. S6 Time-resolved gene regulations and protein expressions in *S. cerevisiae* after LPS exposure.** (A-B) Inferred gene expression patterns (A) and protein expression patterns (B) of yeast cells after LPS exposure were analyzed using the short time sequence expression miner (STEM). Each profile represents a specific pattern of gene or protein clusters enriched. The x-axis indicates the timeline, while the y-axis illustrates the treatment group's expression level in comparison to the control group. The top 5 pathways were obtained via Gene Ontology (GO) enrichment of gene clusters or protein clusters.


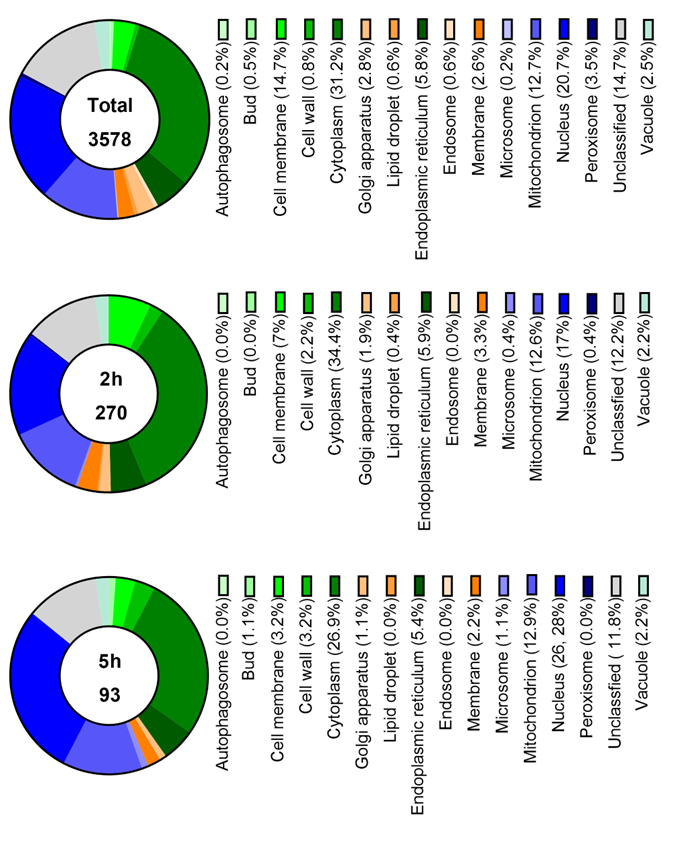


**Fig. S7 Subcellular location of total proteins and DEPs after LPS exposure, based on the UniProt database.**


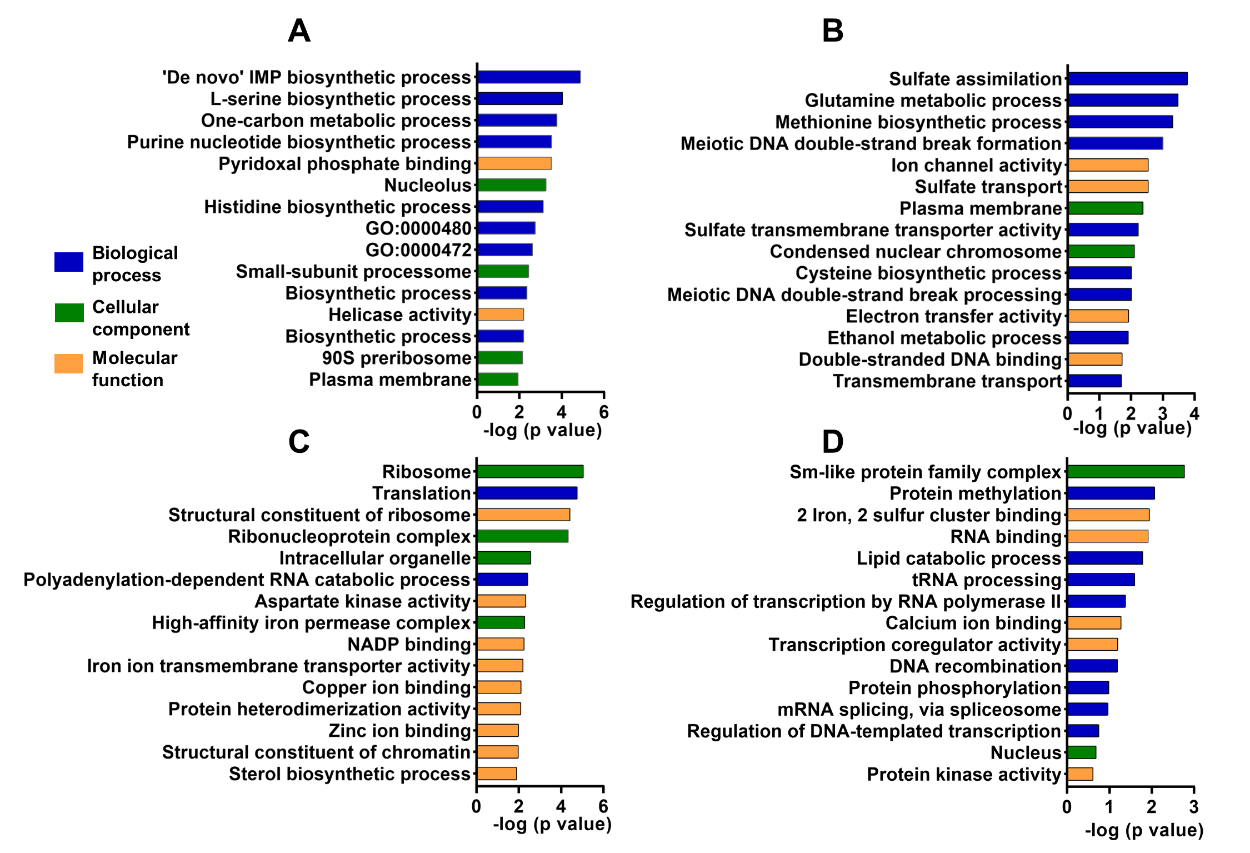


**Fig. S8 The top 15 items enriched in DEGs and DEPs Gene Ontology (GO).** (A) GO enrichment of differentially expressed genes after 2 hours of LPS exposure. GO 0000480 (endonucleolytic cleavage in 5'-ETS of tricistronic rRNA transcript (SSU-rRNA, 5.8S rRNA, LSU-rRNA)). GO0000472 (endonucleolytic cleavage to generate mature 5'-end of SSU-rRNA from (SSU-rRNA, 5.8S rRNA, LSU-rRNA)). GO0000447 (endonucleolytic cleavage in ITS1 to separate SSU-rRNA from 5.8S rRNA and LSU-rRNA from tricistronic rRNA transcript (SSU-rRNA, 5.8S rRNA, LSU-rRNA)). (B) GO enrichment of differentially expressed genes after 5 hours of LPS exposure. (C) GO enrichment of differentially expressed proteins after 2 hours of LPS exposure. (D) GO enrichment of differentially expressed proteins after 5 hours of LPS exposure.


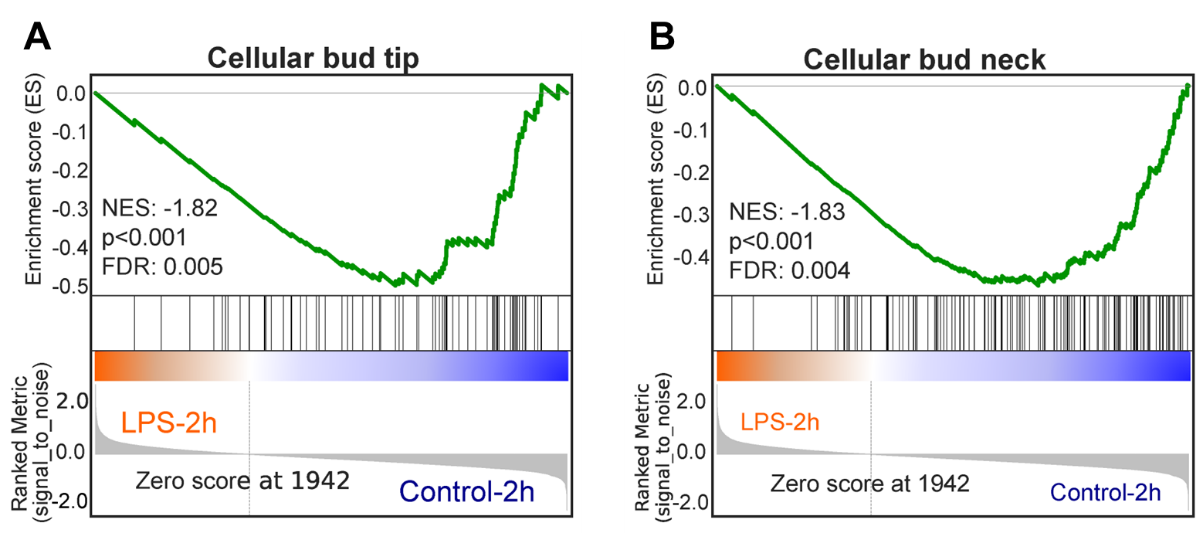


**Fig. S9 The results of transcriptomic GSEA demonstrate the down-regulation of cell budding.** (A) GO term “cellular bud tip”. (B) GO term “cellular bud neck”.


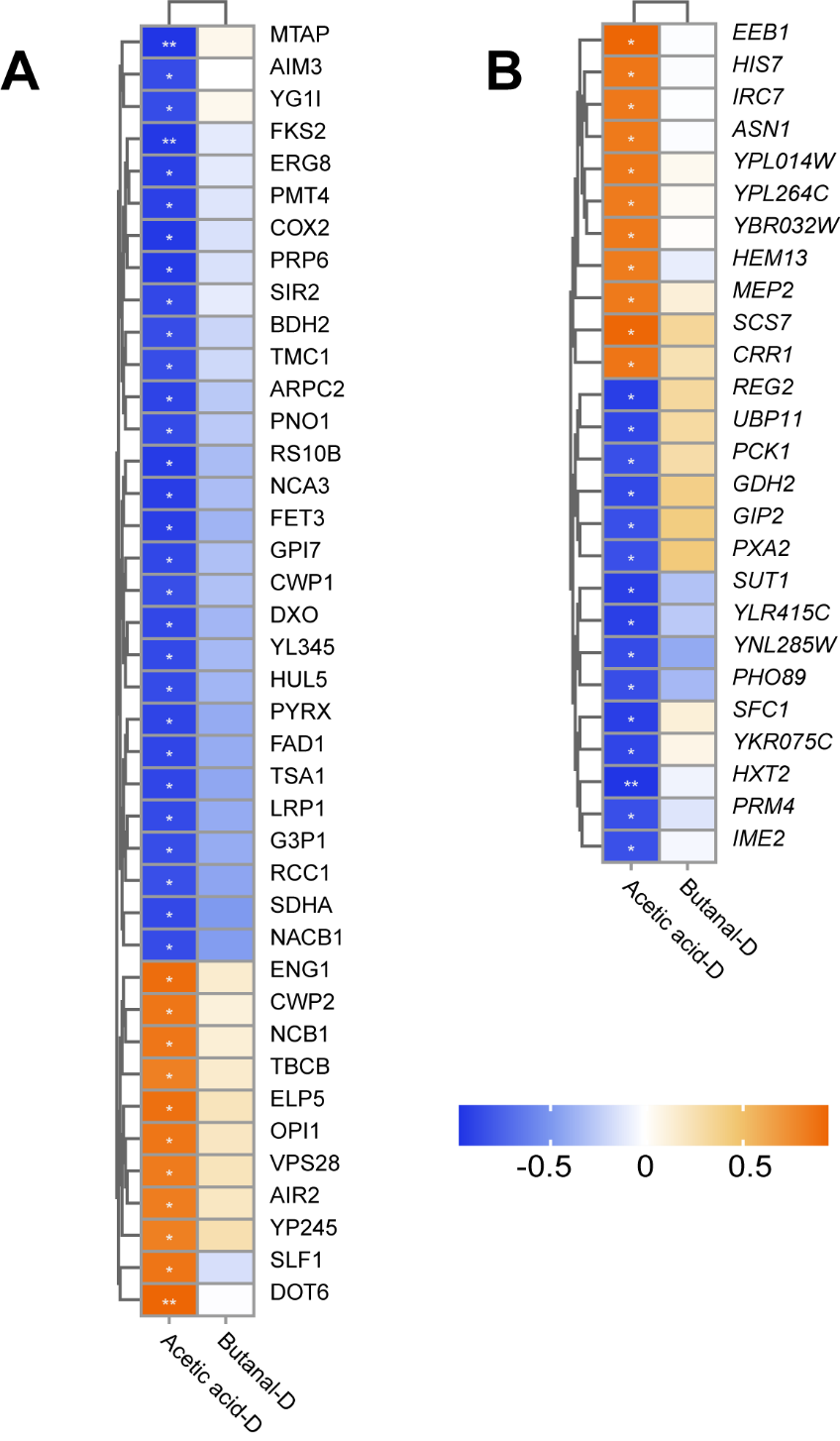


**Fig. S10 Pearson correlation analysis of levels of differentially released VOCs in 2-hour samples with differentially expressed proteins or genes.** (A) Proteins. or (B) Genes. Data include all 6 samples at 2 hours. *, **, *** represent significant correlation at 0.05,0.01,0.001 level respectively. Proteins and genes with 0 expression in any sample have been kicked out.


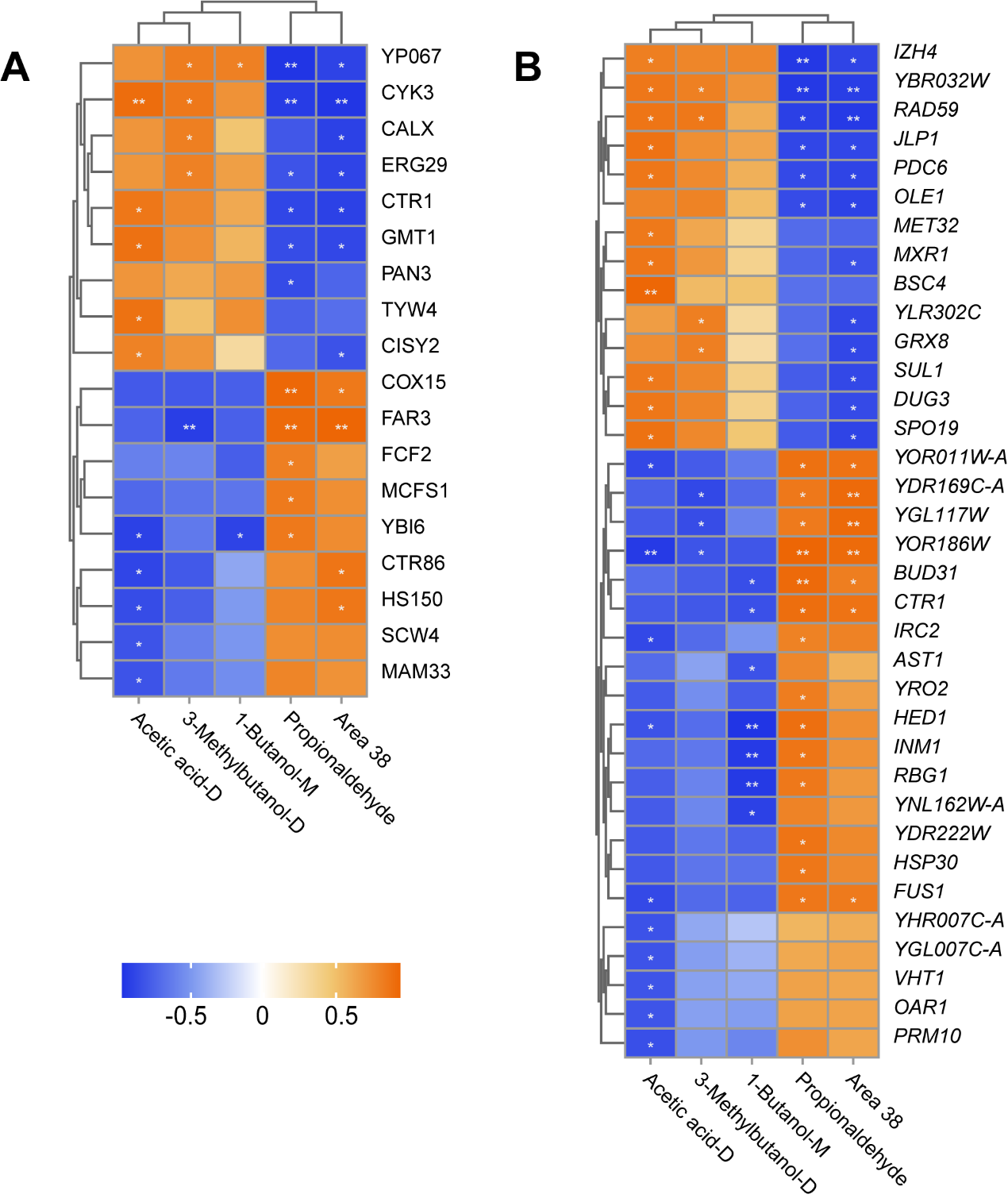


**Fig. S11 Pearson correlation analysis of levels of differentially released VOCs in 5-hour samples with differentially expressed proteins or genes.** (A) Proteins. or (B) Genes. Data include all 6 samples at 5h. *, **, *** represent significant correlation at 0.05,0.01,0.001 level respectively. Proteins and genes with 0 expression in any sample have been kicked out.


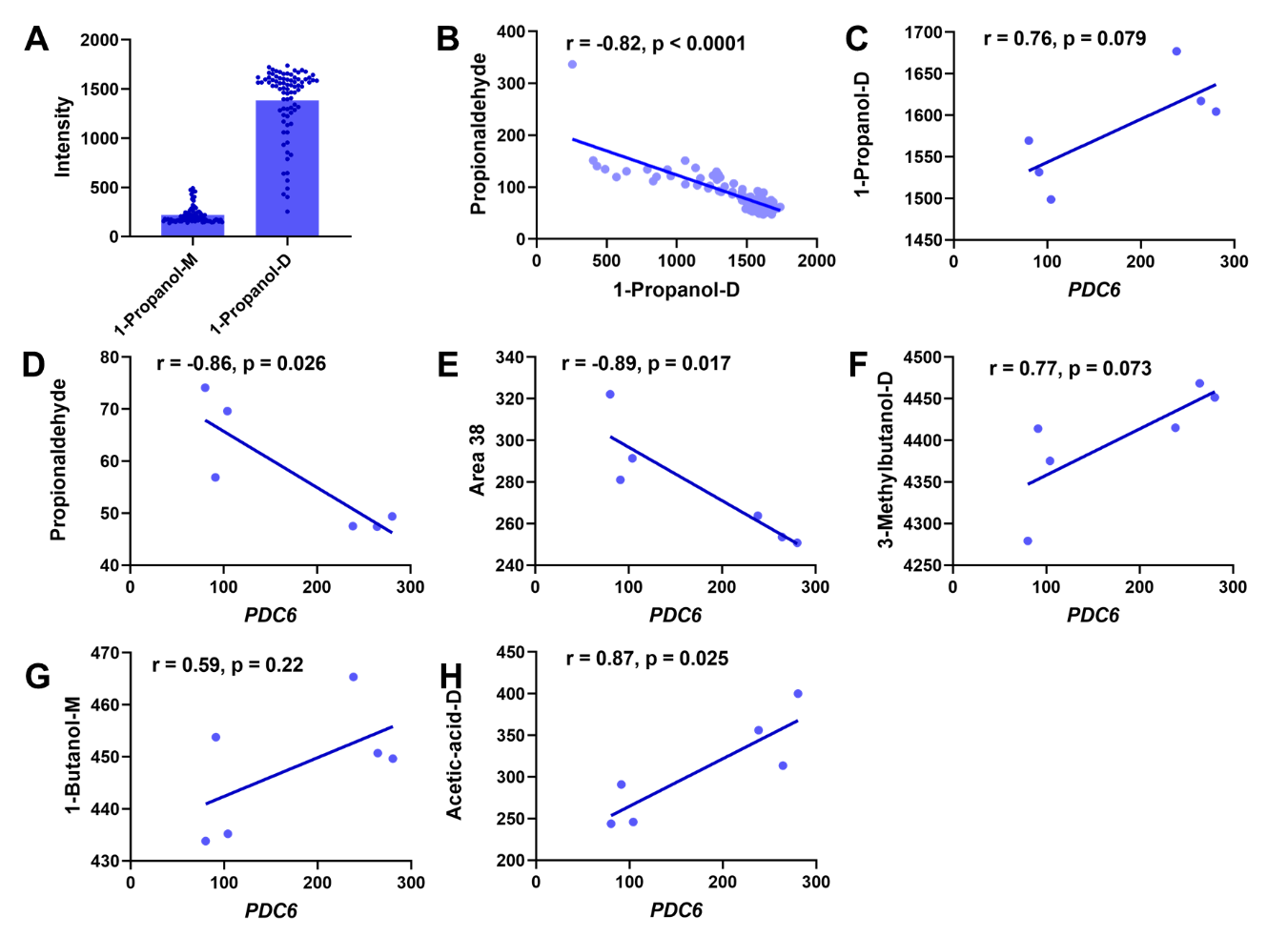


**Fig. S12 Detailed analysis of down-regulated VOCs after 5 hours of LPS treatment.** (A) Comparison of signal intensities of 1-propanol-M and 1-propanol-D in all samples. (B) Pearson correlation analysis of 1-propanol D and propionaldehyde in signal value changes caused by LPS exposure. (C) Pearson correlation analysis between 1-propanol-D level and *PDC6* transcriptomic expression in 5-hour samples. (D) Pearson correlation analysis between propionaldehyde level and *PDC6* transcriptomic expression in 5-hour samples. (E) Pearson correlation analysis between area38 level and *PDC6* transcriptomic expression in 5-hour samples. (F) Pearson correlation analysis between 3-methylbutanol-D level and *PDC6* transcriptomic expression in 5-hour samples. (G) Pearson correlation analysis between 1-butanol-M level and *PDC6* transcriptomic expression in 5-hour samples. (H) Pearson correlation analysis between acetic acid-D level and *PDC6* transcriptomic expression in 5-hour samples.


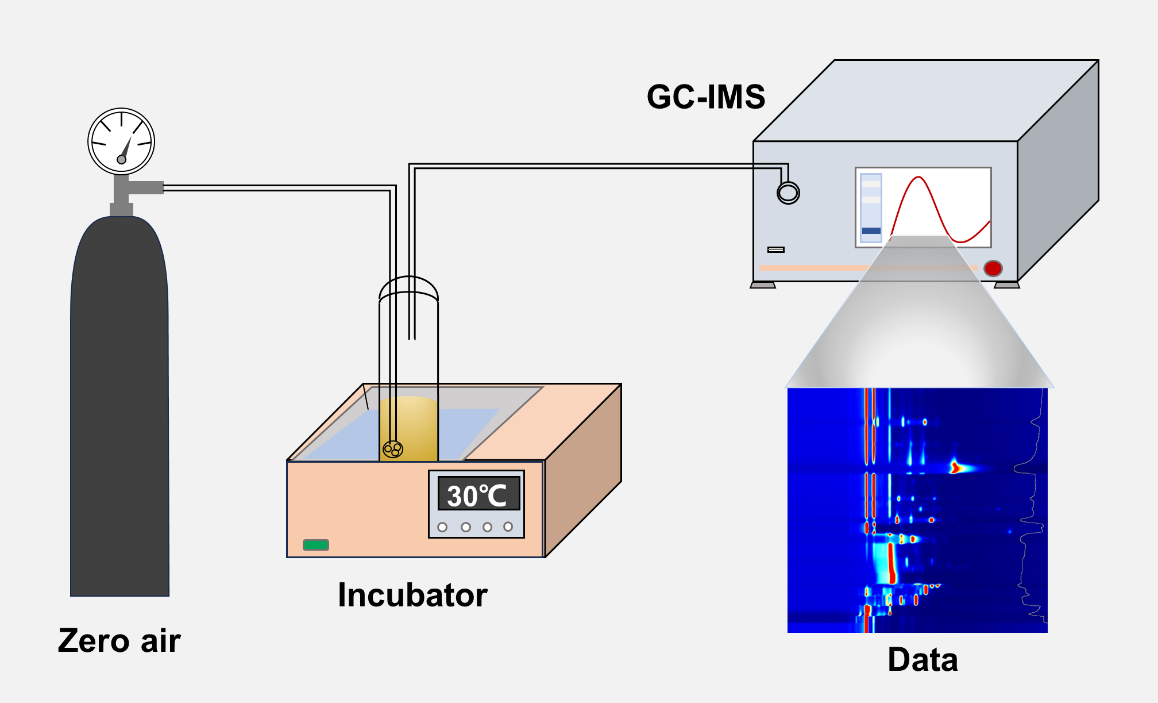


**Fig. S13 Schematic diagram of VOCs online detection.**

## Supplementary Tables

**Table S1.** The retention index, drift time, and qualitative information of 51 VOCs peaks were detected through VOCal software and the GC-IMS library.

| **VOC** | **CAS** | **Retention Index** | **Drift Time** |
| --- | --- | --- | --- |
| Area 1 |  | 1437.8 | 1.42085 |
| Area 2 |  | 1430.7 | 1.54435 |
| Acetic acid-M | 64-19-7 | 1406.1 | 1.05036 |
| Acetic acid-D | 64-19-7 | 1407.4 | 1.15428 |
| Area 5 |  | 1365.3 | 1.16633 |
| Area 6 |  | 1345.5 | 1.23109 |
| Area 7 |  | 1334.2 | 1.48862 |
| Ethyl octanoate-M | 106-32-1 | 1373.8 | 1.47959 |
| Ethyl octanoate-D | 106-32-1 | 1373.6 | 2.03381 |
| 3-Hydroxy-2-butanone-M | 513-86-0 | 1242.9 | 1.07015 |
| 3-Hydroxy-2-butanone-D | 513-86-0 | 1242.3 | 1.33521 |
| Area 12 |  | 1257 | 1.16348 |
| Area 13 |  | 1212.6 | 1.17219 |
| Ethyl hexanoate-M | 123-66-0 | 1196.9 | 1.34143 |
| Ethyl hexanoate-D | 123-66-0 | 1195.4 | 1.80062 |
| 3-Methylbutanol-M | 123-51-3 | 1176 | 1.23815 |
| 3-Methylbutanol-D | 123-51-3 | 1173.7 | 1.49449 |
| 1-Butanol-M | 71-36-3 | 1125.2 | 1.18713 |
| 1-Butanol-D | 71-36-3 | 1128.2 | 1.38996 |
| Isoamyl acetate-M | 123-92-2 | 1106.5 | 1.31157 |
| Isoamyl acetate-D | 123-92-2 | 1107.3 | 1.75582 |
| 2-Methyl-1-propanol-M | 78-83-1 | 1083.6 | 1.17468 |
| 2-Methyl-1-propanol-D | 78-83-1 | 1082.7 | 1.38001 |
| Area 24 |  | 1059.7 | 1.81771 |
| 1-Propanol-M | 71-23-8 | 1040.8 | 1.1081 |
| 1-Propanol-D | 71-23-8 | 1041.1 | 1.25756 |
| Area 27 |  | 977.4 | 1.22524 |
| 2-Pentanone | 107-87-9 | 978.3 | 1.37605 |
| Area 29 |  | 1008.5 | 1.27506 |
| Area 30 |  | 955.3 | 1.2616 |
| 2-Methylbutanal | 96-17-3 | 898.8 | 1.40888 |
| Area 32 |  | 895.8 | 1.37494 |
| Ethyl acetate | 141-78-6 | 873.1 | 1.34225 |
| Butanal-M | 123-72-8 | 821.9 | 1.11596 |
| Butanal-D | 123-72-8 | 826.3 | 1.2819 |
| Propionaldehyde | 123-38-6 | 798.7 | 1.07321 |
| Area 37 |  | 748.9 | 1.13733 |
| Area 38 |  | 904.3 | 1.28316 |
| Ethanol | 64-17-5 | 914.4 | 1.14739 |
| Area 40 |  | 1202.4 | 1.37215 |
| Area 41 |  | 1035.7 | 1.38853 |
| Ethyl butanoate | 105-54-4 | 1035.2 | 1.56334 |
| 4-Methyl-2-pentanone | 108-10-1 | 1012.7 | 1.48413 |
| Area 44 |  | 968.3 | 1.46574 |
| Area 45 |  | 958 | 1.51153 |
| Ethyl isobutyrate | 97-62-1 | 960.7 | 1.56676 |
| Area 47 |  | 960.3 | 1.31622 |
| Area 48 |  | 891.6 | 1.67587 |
| Area 49 |  | 1285.2 | 1.17938 |
| Area 50 |  | 1281.5 | 1.31527 |
| Dimethyl sulfide | 75-18-3 | 756.1 | 0.96514 |

**Table S2.** Intracellular differentially expressed metabolites annotatable by KEGG were found in samples collected after 2 and 5 hours of LPS treatment.

| **2h** | **Intracellular DEMs** | **Dataclass** | **m/z** | **Retention time (min)** | **CAS** | **VIP** | **P-value** | **log2(FC)** | **KEGG** | **ID Annotation** |
| --- | --- | --- | --- | --- | --- | --- | --- | --- | --- | --- |
| 1 | L-Methionine | GC | 176.086 | 13.243 | 63-68-3 | 3.1645 | 0.00591 | 4.76294 | C00073 | sce00270\|sce00970 |
| 2 | Gluconic Acid | GC | 204.087 | 19.387 | 526-95-4 | 1.81325 | 0.0433 | 2.30349 | C00257 | sce00030 |
| 3 | Citrulline | GC | 157.098 | 17.371 | 372-75-8 | 1.79052 | 0.04733 | -1.0419 | C00327 | sce00220 |
| 4 | D-Galactose | GC | 204.111 | 18.09 | 59-23-4 | 1.63704 | 0.02835 | 1.81083 | C00124 | sce00052 |
| 5 | Citric Acid | GC | 273.101 | 17.273 | 77-92-9 | 1.339 | 2.1E-05 | 1.19091 | C00158 | sce00020\|sce00250\|  sce00630 |
| 6 | Sarcosine | GC | 258.113 | 6.831 | 107-97-1 | 1.1343 | 0.00091 | -0.9587 | C00213 | sce00260\|sce00330 |
| 7 | Adenosine monophosphate | LC | 348.07 | 0.76842 | 61-19-8 | 6.5199 | 0.00986 | -0.4697 | C00020 | sce00230 |
| 8 | Jasmonic acid | LC | 233.114 | 7.05453 | 59366-47-1 | 3.36655 | 0.03284 | -0.1101 | C08491 | sce00592 |
| 9 | Xanthine | LC | 153.041 | 1.09277 | 69-89-6 | 2.18834 | 0.01935 | -1.0294 | C00385 | sce00230 |
| 10 | Phthalic acid | LC | 149.023 | 11.2287 | 88-99-3 | 2.07656 | 0.01048 | -0.1151 | C01606 | sce02010 |
| 11 | N-methyl-L-glutamic Acid | LC | 184.058 | 0.56465 | 6753-62-4 | 1.83493 | 0.03217 | -0.1831 | C01046 | sce00680 |
| 12 | LysoPC(18:0/0:0) | LC | 524.371 | 11.6303 | 19420-57-6 | 1.78025 | 0.02454 | -0.4871 | C04230 | sce00564 |
| 13 | Pyridoxal | LC | 185.092 | 0.81313 | 66-72-8 | 1.56761 | 0.02272 | 1.40832 | C00250 | sce00750 |
| 14 | (S)-3-Sulfonatolactate | LC | 192.976 | 0.63088 |  | 1.39307 | 0.00561 | -1.8097 | C11499 | sce00270 |
| 15 | Pseudouridine | LC | 267.058 | 0.81313 | 1445-07-4 | 1.06267 | 0.02001 | -0.4751 | C02067 | sce00240 |
| 16 | D-Lactate | GC | 147.061 | 5.916 | 50-21-5 | 1.08429 | 0.00226 | 0.82276 | C00256 | sce00620 |
| 17 | Tris(2-butoxyethyl) phosphate | LC | 421.232 | 11.0467 | 78-51-3 | 1.57305 | 0.00324 | -0.21 | C14446 |  |
| 18 | D-Threitol | GC | 174.081 | 12.952 | 2418-52-2 | 3.12169 | 1.6E-08 | -6.0353 | C16884 |  |
| 19 | D-Gulose | GC | 204.124 | 19.198 | 50-99-7 | 2.14233 | 0.00774 | 2.909 | C00738 |  |
| 20 | Dodecanol | GC | 243.223 | 13.895 | 112-53-8 | 1.50076 | 0.00681 | -1.3351 | C02277 |  |
| 21 | C16 Sphinganine | LC | 274.274 | 8.42003 |  | 1.23639 | 0.02215 | -0.1343 | C13915 |  |
| 22 | Thyrotropin releasing hormone | LC | 361.163 | 9.09878 | 24305-27-9 | 2.0667 | 0.00516 | -0.4737 | C03958 |  |
| 23 | 3-Phosphoglyceric Acid | GC | 299.07 | 17.164 | 820-11-1 | 1.59433 | 0.02719 | 1.37282 | C00597 |  |
| 24 | 12,13-DHOME | LC | 337.234 | 9.97767 | 7293-40-5 | 14.2764 | 0.00261 | 1.18551 | C14829 |  |
| 25 | Cefadroxil | LC | 364.096 | 3.71005 | 66592-87-8 | 1.8467 | 0.02606 | 1.80985 | C06878 |  |
| 26 | Nicorandil | LC | 192.041 | 15.0847 | 65141-46-0 | 1.00949 | 0.00126 | -0.4905 | C13280 |  |
| 27 | (-)-Perillyl alcohol | LC | 135.117 | 9.97767 | 18457-55-1 | 1.39547 | 0.00799 | 0.77027 | C02452 |  |
| 28 | Sulfanilamide | LC | 217.029 | 0.72162 | 63-74-1 | 10.988 | 0.01131 | 1.88148 | C07458 |  |
| 29 | Metrizoic acid | LC | 672.781 | 11.9865 | 1949-45-7 | 1.62901 | 0.02856 | 0.27421 | C14165 |  |
| 30 | 3-Phosphoglyceric acid | LC | 184.985 | 0.7375 | 820-11-1 | 4.46167 | 0.02151 | -1.0625 | C00597 |  |
| 31 | Sodium sulfate | LC | 164.92 | 0.67675 | 7757-82-6 | 1.98773 | 0.00618 | 2.51457 | C13199 |  |
| 32 | D-Ribose 5-phosphate | LC | 229.011 | 0.7375 | 34980-65-9 | 1.86938 | 0.03853 | -1.5202 | C03736 |  |
| 33 | Pantoprazole | LC | 428.073 | 3.72217 | 102625-70-7 | 1.07174 | 0.03438 | 1.82354 | C11806 |  |
| 34 | Chloroquine | LC | 320.188 | 4.00817 | 54-05-7 | 1.09951 | 0.03123 | -2.7273 | C07625 |  |
| 35 | Necatorine | LC | 527.102 | 0.7375 | 89915-35-5 | 1.79461 | 0.01678 | -2.2837 | C08594 |  |
| 36 | Zingerone | LC | 193.087 | 8.11167 | 122-48-5 | 1.0718 | 0.03972 | -0.0985 | C17497 |  |
| **5h** | **Intracellular DEMs** | **Dataclass** | **m/z** | **Retention time (min)** | **CAS** | **VIP** | **P-value** | **log2(FC)** | **KEGG** | **ID Annotation** |
| 1 | Ornithine | GC | 142.085 | 14.538 | 3184-13-2 | 2.83502 | 0.00156 | 2.01028 | C00077 | sce00220\|sce00330\|  sce00480 |
| 2 | L-Glutamine | GC | 156.067 | 16.782 | 56-85-9 | 1.94057 | 0.00886 | 1.57624 | C00064 | sce00220\|sce00250\|  sce00970\|sce00630\|  sce00910\|sce02010\|  sce00240\|sce00230\|  sce00750 |
| 3 | Citrulline | GC | 157.098 | 17.371 | 372-75-8 | 1.68263 | 0.00141 | 1.02116 | C00327 | sce00220 |
| 4 | Oxalic Acid | GC | 147.055 | 7.038 | 144-62-7 | 1.64081 | 0.00321 | 0.87453 | C00209 | sce00630\|sce00230 |
| 5 | Glycerol 3-Phosphate | GC | 299.072 | 16.552 | 57-03-4 | 1.46292 | 0.0041 | 0.91235 | C00093 | sce02010\|sce00561\|  sce00564 |
| 6 | 4-Aminobutyric Acid | GC | 174.114 | 13.343 | 56-12-2 | 1.41468 | 0.03531 | 0.58691 | C00334 | sce00250\|sce00330\|  sce00410\|sce00650\|  sce00760 |
| 7 | Sarcosine | GC | 258.113 | 6.831 | 107-97-1 | 1.39133 | 0.00126 | -0.7451 | C00213 | sce00330\|sce00260 |
| 8 | L-Fucose | GC | 290.152 | 15.218 | 2438-80-4 | 1.36745 | 0.01472 | 0.72568 | C01019 | sce00051\|sce00520 |
| 9 | L-Glutamic Acid | GC | 246.155 | 14.656 | 56-86-0 | 1.23276 | 0.00311 | 0.60502 | C00025 | sce00220\|sce00250\|  sce00330\|sce00480\|  sce00970\|sce00630\|  sce00910\|sce02010\|  sce00650\|sce00430\|  sce00332\|sce00660\|  sce00340\|sce00860 |
| 10 | Hydroxypropionic Acid | GC | 147.065 | 7.252 | 503-66-2 | 1.21868 | 0.01345 | -0.6644 | C01013 | sce00410\|sce00240\|  sce00640 |
| 11 | L-Asparagine | GC | 115.049 | 13.096 | 70-47-3 | 1.02814 | 0.01646 | 0.48334 | C00152 | sce00250\|sce00970\|  sce00460 |
| 12 | Pyroglutamic Acid | GC | 156.11 | 13.42 | 98-79-3 | 1.02766 | 0.00333 | 0.42374 | C01879 | sce00480 |
| 13 | Aminoparathion | LC | 242.04 | 5.3848 | 3735-01-1 | 1.16951 | 0.01809 | 0.3004 | C06605 |  |
| 14 | Bis(2-ethylhexyl) phthalate | LC | 391.283 | 14.5335 | 117-81-7 | 3.19968 | 0.00371 | -0.3101 | C03690 |  |
| 15 | Hydroxyhydroquinone | GC | 342.147 | 14.375 | 533-73-3 | 1.03046 | 0.04659 | -0.4443 | C02814 |  |
| 16 | Benzaldehyde | LC | 107.049 | 2.26767 | 100-52-7 | 1.19796 | 0.00067 | -0.242 | C00261 |  |
| 17 | Cefdinir | LC | 376.018 | 5.3848 | 91832-40-5 | 1.05067 | 0.02456 | 0.30988 | C08110 |  |
| 18 | Oryzalin | LC | 369.084 | 10.3015 | 19044-88-3 | 2.46535 | 0.03858 | 1.11386 | C18877 |  |

**Table S3.** Qualitative information and statistical analysis of amino acid substances identified in the intracellular metabolomic. The p value is a t- test performed on each amino acid signal value of the control group and the treatment group.

| **Metabolites** | **CAS** | **2h** | | | **5h** | | |
| --- | --- | --- | --- | --- | --- | --- | --- |
|  |  | **VIP** | **P value** | **FC** | **VIP** | **P value** | **FC** |
| Sarcosine | 107-97-1 | 1.134 | 0.001 | 0.515 | 1.391 | 0.001 | 0.597 |
| Pyroglutamic Acid | 98-79-3 | 0.203 | 0.309 | 0.943 | 1.028 | 0.003 | 1.341 |
| Pipecolic Acid | 535-75-1 | 1.2 | 0.16 | 0.038 | 1.02 | 0.341 | 0.108 |
| Ornithine | 3184-13-2 | 0 | 0.11 | 1 | 2.835 | 0.002 | 4.029 |
| L-Valine | 72-18-4 | 0.682 | 0.257 | 2.281 | 0.53 | 0.099 | 1.235 |
| L-Tyrosine | 60-18-4 | 0.644 | 0.21 | 2.399 | 0.069 | 0.563 | 1.076 |
| L-Threonine | 72-19-5 | 0.449 | 0.036 | 0.878 | 0.61 | 0.1 | 1.176 |
| L-Serine | 56-45-1 | 0.722 | 0.048 | 0.701 | 0.818 | 0.044 | 1.274 |
| L-Proline | 147-85-3 | 0.735 | 0.004 | 0.734 | 0.812 | 0.029 | 1.275 |
| L-Phenylalanine | 63-91-2 | 0.639 | 0.217 | 2.327 | 0.289 | 0.251 | 1.136 |
| L-Methionine | 63-68-3 | 3.165 | 0.006 | 27.151 | 0.74 | 0.174 | 1.239 |
| L-Lysine | 56-87-1 | 0.418 | 0.138 | 0.877 | 0.145 | 0.762 | 1.03 |
| L-Leucine | 61-90-5 | 0.767 | 0.176 | 2.345 | 0.5 | 0.115 | 1.177 |
| L-Isoleucine | 73-32-5 | 0.615 | 0.187 | 2.059 | 0.371 | 0.154 | 1.12 |
| L-Homocysteine | 6027-13-0 | 1.216 | 0.159 | 24.2 | 0.155 | 0.535 | 1.06 |
| L-Glutamine | 56-85-9 | 0 | 0.148 | 1 | 1.941 | 0.009 | 2.982 |
| L-Glutamic Acid | 56-86-0 | 0.345 | 0.377 | 0.85 | 1.233 | 0.003 | 1.521 |
| L-Aspartic Acid | 56-84-8 | 0.591 | 0.067 | 1.363 | 0.925 | 0.018 | 1.361 |
| L-Asparagine | 70-47-3 | 1.2 | 0.165 | 0.398 | 1.028 | 0.016 | 1.398 |
| L-Alanine | 56-41-7 | 0.292 | 0.41 | 0.874 | 0.804 | 0.043 | 1.253 |
| L-2,4-Diaminobutyric Acid | 1758-80-1 | 0.593 | 0.341 | 0.118 | 0 | 0.427 | 1 |
| Glycine | 56-40-6 | 0.788 | 0.003 | 0.713 | 0.899 | 0.024 | 1.32 |
| D-Threonine |  | 1.055 | 0.338 | 0.024 | 0.618 | 0.097 | 1.182 |
| D-Phenylalanine | 150-30-1 | 0.861 | 0.169 | 3.345 | 1.873 | 0.172 | 0.647 |
| Dl-Homoserine | 1927-25-9 | 0.421 | 0.233 | 0.836 | 0.207 | 0.659 | 0.957 |
| Citrulline | 372-75-8 | 1.791 | 0.047 | 0.486 | 1.683 | 0.001 | 2.03 |
| Alpha-Aminobutyric Acid |  | 1.355 | 0.217 | 0.681 | 0.822 | 0.021 | 1.261 |
| Alanylalanine | 1948-31-8 | 0.131 | 0.651 | 1.088 | 0.507 | 0.345 | 0.89 |
| Ala-Gly | 687-69-4 | 0.051 | 0.731 | 0.995 | 0.423 | 0.003 | 1.05 |
| 5-Amino-Pentanoic Acid | 660-88-8 | 0.197 | 0.376 | 1.063 | 0.118 | 0.536 | 1.02 |
| 4-Aminobutyric Acid | 56-12-2 | 0.883 | 0.246 | 0.375 | 1.415 | 0.035 | 1.502 |
| 3-Amino-2-Piperidone | 1892-22-4 | 0.375 | 0.185 | 0.865 | 1.332 | 0.005 | 1.691 |
| 2s-Amino-Pentanoic Acid |  | 0.25 | 0.42 | 1.193 | 0.624 | 0.042 | 1.188 |
| 2r-Amino-Butanoic Acid | 2623-91-8 | 0.325 | 0.071 | 0.916 | 0.174 | 0.637 | 1.02 |
| 2,5-Diaminopentanoic Acid | 70-26-8 | 0.84 | 0 | 0.716 | 0.948 | 0.014 | 1.404 |
